# Targeting cancer-associated adipocyte-derived CXCL8 inhibits triple-negative breast cancer progression and enhances the efficacy of anti-PD-1 immunotherapy

**DOI:** 10.1038/s41419-023-06230-z

**Published:** 2023-10-28

**Authors:** Renhong Huang, Zheng Wang, Jin Hong, Jiayi Wu, Ou Huang, Jianrong He, Weiguo Chen, Yafen Li, Xiaosong Chen, Kunwei Shen

**Affiliations:** Department of General Surgery, Comprehensive Breast Health Center, Ruijin Hospital, Shanghai Jiao Tong University School of Medicine, 197 Ruijin Second Road, 200025 Shanghai, China

**Keywords:** Chemokines, Biomarkers

## Abstract

Cancer-associated adipocytes (CAAs), one of the primary stromal components, exhibit intimate crosstalk and release multiple cell factors mediating local and systemic biological effects. However, the role of CAAs in the regulation of systemic immune responses and their potential value in the clinical treatment of triple-negative breast cancer (TNBC) are not well described. Transcriptome sequencing was performed on CAA and normal adipocyte (NA) tissues isolated from surgically resected samples from TNBC patients and healthy controls. Cytokines, including C-X-C motif chemokine ligand 8 (CXCL8, also known as IL-8), secreted from NAs and CAAs were compared by transcriptome sequencing and enzyme-linked immunosorbent assay (ELISA). Proliferation, migration and invasion assays were employed to analyze the role of CAAs and CAA-derived CXCL8 (macrophage inflammatory protein-2 (MIP2) as a functional surrogate in mice). TNBC syngraft models were established to evaluate the curative effect of targeting CXCL8 in combination with anti-PD-1 therapies. Real-time quantitative polymerase chain reaction (RT-qPCR), western blotting (WB), polymerase chain reaction (PCR) array, flow cytometry, immunohistochemistry (IHC), and immunofluorescence (IF) were applied to analyze immune cell infiltration and epithelial–mesenchymal transition (EMT) markers. Specifically, we demonstrated that CAAs and CAA-derived CXCL8 played important roles in tumor growth, EMT, metastasis and tumor immunity suppression. CAA-derived CXCL8 remodeled the tumor immune microenvironment not only by suppressing CD4^+^ T and CD8^+^ T immune cell infiltration but also by upregulating CD274 expression in TNBC. The combination of targeting the CXCL8 pathway and blocking the PD-1 pathway synergistically increased the tumor immune response and inhibited tumor progression. Thus, our results highlight the molecular mechanisms and translational significance of CAAs in tumor progression and immune ecosystem regulatory effects and provide a better understanding of the potential clinical benefit of targeting CAA-derived CXCL8 in antitumor immunity and as a new therapeutic moiety in TNBC.

## Introduction

Breast cancer (BC) is the most prevalent tumor in females and has a relatively high mortality worldwide [1]. Among the different molecular types of BC, triple-negative breast cancer (TNBC) is an aggressive subtype and has the worst prognosis due to a lack of well-defined and effectively treatable targets [2, 3]. Beyond the use of conventional therapeutics for TNBC, such as chemotherapy, the past few years have witnessed a rapid revolution in the applications of antibody-based immunotherapies to modulate immune responses against tumors [4–6]. Indeed, established candidate biomarkers, such as CD274 and CTLA4, have provided an attractive approach to release the brake on antitumor immunity [7–9]. It is well known that the tumor immune microenvironment (TIME) contains numerous tumor-infiltrating lymphocytes (TILs) and plays an indispensable role in antitumor immunity and tumor progression in TNBC [10–12]. Unfortunately, the obvious benefits of antitumor immunotherapy remain limited to a small subset of individuals with TNBC [13, 14]. These limitations must be resolved to efficiently induce the host antitumor immune response [15]. Admittedly, how to increase immune cell infiltration and immunotherapy efficacy by conquering tumor-induced immune suppression still needs to be further investigated.

Indeed, the immune system exerts indispensable roles in tumorigenesis and tumor progression [16, 17]; however, the complex molecular mechanisms regulating tumor immunity and the tumor microenvironment (TME) are not completely understood. In TNBC, the TME is a highly heterogeneous ecosystem that contains multiple infiltrating immune cells, fibroblasts, and matrix components that promote tumor migration, invasion, metastasis and even drug resistance [18–22]. Amid the TME of TNBC, cancer-associated adipocytes (CAAs), a type of tumor-educated adipocyte, participate in crosstalk with breast cancer and are capable of secreting various cytokines, adipokines and chemokines, which subsequently lead to changes in cancer cell phenotype and function [23, 24]. On the one hand, emerging evidence has demonstrated that CAAs facilitate BC cell proliferation, migration, and invasion [25–27]. On the other hand, CAAs also intimately interact with immune cells and regulate systemic inflammation given that adipose tissue serves as the primary source of many proinflammatory cytokines in obesity [28, 29]. However, the mechanism by which CAAs function in reprogramming the immune status and transferring this information to TNBC cells to stimulate site-specific antitumor immune responses is currently unknown.

Notably, the PD-1/CD274 checkpoint is a critical mediator of immunosuppression in the TIME, and immune checkpoint inhibitors (ICIs) represent promising therapeutic targets for TNBC patients [7, 21, 30, 31]. Previous data have indicated that cytokines, such as IL2, IL6, TGF-β, and CXCL5, in an immune-reactive TME, may stimulate CD274 expression on tumor cells through distinct signaling, potentially representing one of the causes of immune tolerance [32–35]. Here, it is uncertain whether CAAs could play such a role in linking their secreted cytokines to cancer-tumor immunity, their capacity to exhibit tumor progression, and their clinical therapeutic value in targeted therapy. In this study, we aimed to analyze the transcriptomic and secretory profiles of CAAs and investigate their biological mechanisms in TNBC. Moreover, we aimed to unravel the molecular interactions between TNBC cells and CAAs and to identify novel therapeutic targets against CAAs to sensitize TNBC cells to ICIs.

## Methods

### Patients and adipocyte tissue samples

CAA and normal adipocyte (NA) samples were isolated from 10 TNBC patients and 10 healthy controls who underwent breast surgery between September 2020 and September 2022 at Comprehensive Breast Health Center, Ruijin Hospital, Shanghai Jiao Tong University School of Medicine (Supplementary Table S1). Collected samples were subjected to immunohistochemistry (IHC) and immunofluorescence (IF), adipocyte isolation and cell culture, which are described below in detail. All the enrolled participants or their guardians signed informed consent forms, and the procedures were approved by the Ethical Committee of Ruijin Hospital, Shanghai Jiao Tong University School of Medicine.

### Isolation, characterization, and differentiation of adipocytes

NA and CAA isolation was approved by the Ethical Committee of Ruijin Hospital, Shanghai Jiao Tong University School of Medicine, and informed consent was obtained from all the patients. Adipocytes represent an indispensable component of the BC tumor microenvironment. To investigate the possible role of CAAs, we isolated and cultured NAs and CAAs in normal adipocytes and TNBC adipocyte tissues. NAs and CAAs adjacent to the tumor (within 2 cm) were dissociated from derived breast begin patients and TNBC patients, respectively. The culture process was described as previously reported [36]. Briefly, adipocytes were isolated, washed with PBS, cut into pieces, and digested with type II collagenase (Sigma‒Aldrich, USA) at 37 °C in a shaking bath for 30 min. Subsequently, the digested liquid was quenched with DMEM/F12 (Gibco, USA) supplemented with 10% FBS (Gibco, USA). Next, a 40-μm blue sterile nylon cell strainer (BD, USA) was applied to filter the suspended samples. The SVFs were plated and then cultured in DMEM/F12 supplemented with 10% FBS, 1% penicillin/streptomycin and 1 mM L-glutamine. Primary preadipocytes were cultured in a cocktail containing 5 μg/ml insulin (Eli Lilly, USA), 1 μM dexamethasone (Sigma‒Aldrich, USA), and 0.5 mM IBMX (Sigma‒Aldrich, USA) for 4 days followed by medium with 5 μg/ml insulin for another 4 days to promote differentiation into mature adipocytes. The polyhedral morphology of differentiated mature adipocytes was assessed based on staining in oil red O solution for 30 min. The detailed steps are illustrated in Fig. 2A.

### Cell culture

MDA-MB-231 and 4T1 cells obtained from the Type Culture Collection of the Chinese Academy of Sciences (Shanghai, China) were generally cultured in high-glucose DMEM and RPMI-1640 medium supplemented with 10% FBS and 1% penicillin/streptomycin, respectively. The cells were maintained in a humidified incubator at 37 °C with 5% CO_2_.

### Coculture assay

A coculture assay was performed as described in our previous study [37]. Briefly, a 6-well Transwell system with 0.4-μm pore polycarbonate membranes (Corning) was applied in the study. CAAs or 3T3L1 cells at a concentration of 1 × 10^5^ cells/well were seeded into the upper chambers and then cocultured with MDA-MB-231 or 4T1 cells (1 × 10^5^ cells/well) in the lower chambers for 7 days.

### Plasmids

The GFP vectors were obtained from the DNA library of Shanghai Jiao Tong University School of Medicine. In accordance with the guidance of the product manual, lentivirus was used to cotransfect the GFP vector, psPAX2 or PMG.2G into HEK293T tool cells to obtain a GFP overexpression lentivirus, which was applied to construct MDA-MB-231, NA or CAA cells stably expressing GFP.

### Cell viability assay and clone formation assays

MDA-MB-231 and 4T1 cells were seeded at 5 × 10^3^ cells per well in a solid 96-well plate and allowed to attach overnight at 37 °C with humidified 5% CO_2_. MDA-MB-231 and 4T1 cells were treated with different concentration gradients of human CXCL8 (Cat #94853, CST) and recombinant mouse MIP2 (Cat#ab243752, Abcam) for 1 week, respectively. Cells were detected using the CellTiter-Glo® Luminescent Cell Viability Assay (Promega, G7573). Briefly, 50 μl CellTiter-Glo was added to the cells in each well, and then the plate was shaken and incubated at room temperature for 30 min to stabilize the luminescent signal. The luminescence of the cells was measured using a Perkin Elmer plate reader. For the clone formation assay, 500 cells were seeded onto 6-well plates. The cells were observed daily under a microscope until the number of cells in most clones was greater than 50. The cells were stained with 0.2% crystal violet for 20 min and then photographed.

### Cell migration, invasion, and wound healing assays

To analyze the role of NA or CAA cell supernatant in the biological function of BC cells, MDA-MB-231 cells were cultured with 50% NA or CAA cell supernatant plus 50% DEME medium containing 2% FBS for 1 week. Similarly, 4T1 cells were cultured with 50% 3T3L1 supernatant plus 50% RAPI-1640 medium containing 2% FBS for 1 week. A wound healing assay was employed to analyze cell migration. Transwell assays were used to analyze cell migration and invasion ability. The detailed procedures have been described in a previous study [38].

### Flow cytometry

The removed tumors were digested into single-cell suspensions and then stained with specific antibodies against cell surface markers (Supplementary Fig. S2). Briefly, cells in suspension were incubated on ice for 30 min and then resuspended in PBS supplemented with 2% FBS. The cells were permeabilized using Fixation/Permeabilization Concentrate (eBioscience) on ice for 30 min and then centrifuged at 1500 rpm at 4 °C for 4 min. Subsequently, the cells were incubated with specific antibodies for 1 h in the dark at 4 °C followed by washing with PBS and pelleting by centrifugation at 1500 rpm for 4 min. The primary antibodies used for flow cytometry are listed in Supplementary Table S4. Ultimately, the cell precipitate was resuspended in paraformaldehyde in buffer and detected by a Beckman Gallios flow cytometer. The flow cytometry data were analyzed using FlowJo software (version 10.1R5, Tree Star, San Carlos, CA, USA).

### Bulk RNA sequencing

Bulk RNA sequencing was performed with the assistance of Novogene Co., Ltd. (Shanghai, China). Differentially expressed transcripts (DETs) were determined using the MA-plot-based method with a random sampling model in the DEGseq package. The thresholds for determining DETs were *P* < 0.05 and an absolute fold change ≥2. Then, DETs were chosen for functional and signaling pathway enrichment analyses using the Gene Ontology (GO) and Kyoto Encyclopedia of Genes and Genomes (KEGG) databases. Significantly enriched pathways were noted when *P* < 0.05, and at least two affiliated genes were included. In addition, different types of immune cells were detected using cell type identification by estimating relative subsets of RNA transcripts (CIBERSORT). Based on the significant CIBERSORT and immune scores based on these genes, we constructed an immune score to compare the scores between the different groups.

### Western blot

Protein was extracted by RIPA reagents with 1% phenylmethanesulfonyl fluoride (PMSF). The concentration of the lysate protein was measured using a BCA protein assay. The protein was separated with sodium dodecyl sulfate‒polyacrylamide gel electrophoresis (SDS‒PAGE) and transferred onto polyvinylidene difluoride membranes (PVDF). Next, the PVDF membranes were blocked with 5% bovine serum albumin (BSA) at room temperature for 1 h. The membranes were incubated with primary antibodies at 4 °C overnight. The membranes were incubated with goat anti-rabbit secondary antibody at room temperature for 1 h. The primary antibodies used for western blotting are listed in Supplementary Table S4. The membranes were detected using a Luminescent Image Analyzer detection system (Fujifilm, LAS-4000).

### RT‒qPCR and RT^2^ profiler PCR array

Total RNA was isolated using TRIzol reagent (Invitrogen, Carlsbad, CA, USA). For the analysis of mRNAs, reverse transcription was performed using the PrimeScriptTM RT reagent kit (Cat# 4368813, Thermo Fisher Scientific) with random primers. The expression of RNAs was assessed by real-time quantitative polymerase chain reaction (RT-qPCR) in triplicate with SYBR Green Master Mix (Cat# 24759100, Roche) on an Applied Biosystems QuantStudio^TM^ Real-Time PCR System. The primers used in the study are listed in Supplementary Table S5. To analyze the immune signatures stimulated by MIP2, an 84-gene RT^2^ profiler polymerase chain reaction array (PCR array) was applied (Supplementary Tables S2 and S3). Comparative cycle threshold values (2^−^^ΔΔCt^) were adopted to analyze the results.

### Cell and tissue immunofluorescence

Cells were seeded on coverslips at a density of 1 × 10^6^ cells per well. The tissue sections were deparaffinized, rehydrated, and immersed in PBS. Antigens were retrieved by hydrolytic autoclaving in the retrieval solution for 15 min at 121 °C. The coverslips or sections were rinsed with PBS and fixed with 4% paraformaldehyde for 20 min. Then, cells were permeabilized in 0.2% Triton X-100 for an additional 20 min. Next, the coverslips were blocked with 5% BSA for 1 h. For cell immunofluorescence, we stained the cells with primary antibody. The antibody information for immunofluorescence is listed in Supplementary Table S4. Then, the cells or tissues on the coverslips were incubated with goat anti-rabbit or goat IgG secondary antibody for 1 h at room temperature in the dark. DAPI was used to stain the cell nuclei. Subsequently, the stained cells were photographed with a fluorescence microscope.

#### Establishment of a mouse model and combined therapy

Female wild-type BALB/c mice (6 weeks old) and female nude mice (6 weeks old) were purchased from Shanghai SLAC Co., Ltd. (Shanghai, China). All procedures involving the mice were approved by the Institutional Animal Care and Use Committee of Shanghai Jiao Tong University School of Medicine (Shanghai, China). To analyze the role of NAs or CAAs in MDA-MB-231 cells in vivo, a suspension containing 1.0 × 10^6^ MDA-MB-231 cells per mouse with or without 2.5 × 10^5^ NAs or CAAs was injected subcutaneously into nude mice. Similarly, subcutaneous tumors were established in female BALB/c mice by implantation in a suspension mix with 1.0 × 10^6^ 4T1 cells per mouse with or without 2.5 × 10^5^ 3T3L1 cells. In addition, we cocultured 4T1 cells with or without MIP2 (20 ng/ml) for 7 days and then established a subcutaneous tumor model to investigate the combined treatment effect. In addition, 4T1 cells (1.0 × 10^6^ cells/mouse) were subcutaneously implanted into BALB/c mice to investigate the effect of combined therapy. When the tumor size reached 50 mm^3^, anti-PD-1 antibody (10 μg once) and anti-MIP2 antibody (10 μg once) were injected every 2 days. The tumors of nude mice in the seventh week and BALB/c mice in the fourth week were harvested by surgical resection based on a margin of gross normal tissue. The lung tissues and subcutaneous tumors were harvested for HE, IF or IHC staining. Schematic diagrams were applied to illustrate the mice model construction process (Supplementary Fig. S1).

### Hematoxylin and eosin (HE) staining

The lungs of mice were stained using an HE staining kit (Beyotime Biotechnology, Shanghai, China). Tissues were fixed with 4% paraformaldehyde for 24 h and dehydrated with an ethanol gradient. The tissues were cleared with xylene, embedded in paraffin, cut into 5-μm sections, dewaxed, dehydrated, and stained with hematoxylin for 5 min and eosin for 3 min. A microscope was applied to record the morphology of the lung metastatic nodules.

### Immunohistochemistry (IHC)

Sectioned tumor tissues of the homograft model were embedded in paraffin, rehydrated, and blocked by incubation with goat serum. Sections were incubated with primary antibody at 4 °C overnight. The antibody information is provided in Supplementary Table S4. Then, the sections were incubated with an HRP-conjugated goat anti-rabbit or mouse secondary antibody at room temperature for 1 h. The sections were treated with the Metal Enhanced DAB Substrate Kit (Dako, Denmark, EU) and stained with hematoxylin (Beyotime, China). The density of target proteins was measured by calculating integrated optical density (IOD) using ImageJ software.

### Cytokines/chemokines detection

Secreted proteins, including TNF-α, IL-1β, IL-2, TGF-β, GM-CSF, IL-17A, TREM2, serpin E1/PAI-1, G-CSF, CCL18, and MMP-8 (all R&D systems, Minneapolis, MN, USA), were measured in the Nas or CAAs supernatants using sandwich ELISA in accordance with the methods provided by the manufacturer’s instructions. The detected signals were visualized using a microarray laser scanning system (GenePix, USA).

### Establishment of patient-derived organoid (PDO)

Human TNBC organoids were stored in a biobank in accordance with a previously published protocol [39]. The regents used in the cell culture of PDO and the detailed protocol was performed according to previously published studies [40].

### Statistical analysis

All the experiments in this study were performed at least in triplicate, and the data are expressed as the means ± SDs. Statistical analysis of the data among the groups was performed using Student’s *t-*test, one-way ANOVA, or two-way ANOVA. GraphPad Prism software (GraphPad Software, Inc.; Los Angeles, CA, USA; version 6.02) and SPSS statistics software (IBM Corporation; Armonk, NY, USA; version 21.0) were used to perform statistical analyses. A *P-*value less than 0.05 was considered significant.

## Results

### CAA was positively associated with the immunosuppressive microenvironment in TNBC patients

**Fig. 1:**
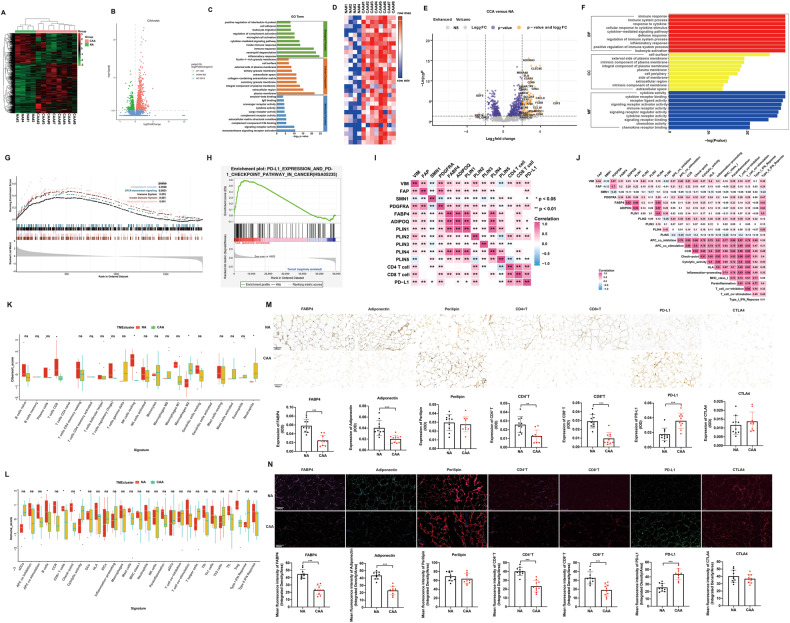
CAA is associated with an immunosuppressive microenvironment in TNBC. **A** Heatmap depicting differential transcriptomic expression with RNA-seq data in 10 normal adipocyte tissues and 10 cancer-associated adipocyte tissues. **B** Volcano plots showing DEGs of NAs and CAAs. **C** GO enrichment scatter plot for upregulated genes in CAAs compared with NAs. **D** Heatmap showing the immune-related gene signature based on RNA-seq data from 10 NA and 10 CAA tissues. **E** Volcano plots represent differential immune-related gene expression in NAs and CAAs. **F** GO mapping of different immune-related genes. **G** Chord diagram clustered by colors showing the relationship between immune-related genes and GO terms of molecular functions. **H** GSEA enrichment of the signaling pathways for immune-related genes. **I**, **J** Data from the TCGA database showing the correlations between immune-related genes or pathways and markers associated with adipose differentiation. **K** The CIBERSORT algorithm was applied to assess differential immune cell infiltration in NAs and CAAs. **L** Comparisons of immune scores in NAs and CAAs. **M**, **N** Representative images of IHC and IF staining and statistical analysis of markers associated with adipose differentiation and immune cells. CAA cancer-associated adipocyte, CIBERSORT Cell-type Identification by Estimating Relative Subsets of RNA Transcript, DEGs differentially expressed gene, GO Gene Ontology, GSEA gene set enrichment analysis, GSVA gene set variation analysis, IF immunofluorescence, IHC immunohistochemistry, KEGG Kyoto Encyclopedia of Genes and Genomes, NA normal adipocyte, TCGA The Cancer Genome Atlas, TNBC triple-negative breast cancer. Data are presented as the means ± SD of at least three independent experiments. *P < 0.05, **P < 0.01, ***P < 0.001.

To systematically discover different gene targets that were aberrantly expressed in CAA compared with NA, we first performed bulk RNA sequencing using 4 NA and 8 CAA tissue samples. Obviously, a distinct difference in the transcriptome level was noted. In total, 1326 genes were upregulated, whereas 803 genes were downregulated in CAAs compared with NAs (Fig. 1A, B). To further analyze the functionality of the differentially expressed genes (DEGs) based on RNA-seq data, GO and KEGG analyses were conducted. GO analysis showed that the upregulated DEGs were significantly associated with the cytokine-mediated signaling pathway and immune response in the biological process (BP) term and cytokine activity and transmembrane signaling receptor activation in the molecular function (MF) term (Fig. 1C). In addition, KEGG pathway analysis revealed that the DEGs were mainly enriched in the immune system (Supplementary Fig. S3A). Moreover, the top 20 enriched REACTOME pathways for the upregulated genes were involved in the immune system and cytokine signaling in the immune system (Supplementary Fig. S3B).

To further investigate the potential impact of CAA on the immune microenvironment, we analyzed the immune-related gene signatures separately (Fig. 1D, E). GO analysis revealed that the upregulated DEGs were associated with the immune response and the immune system in the biological process (BP) term and cytokine activity and immune receptor activity in the molecular function (MF) term (Fig. 1F). Moreover, the cytokine‒cytokine receptor interaction pathway was highly activated in KEGG pathway analysis (Fig. 1G). GO and KEGG enrichment scatter plot for the top 30 upregulated genes also imply similar results (Supplementary Fig. S4). A chord diagram of GO terms revealed that the DEGs were mostly enriched in the regulation of immune receptor activity, receptor-ligand activity, cytokine activity, cytokine receptor binding, and cytokine binding, indicating that these DEGs were closely related to cytokines and an intensive immune phenotype. GSEA also indicated that the immune system was stimulated in the DEGs of the immune signature (Supplementary Fig. S5). Moreover, Gene set variation analysis (GSVA) indicated that the CD274 checkpoint pathway was enriched in CAAs compared with NAs (Fig. 1H).

Based on biological data from the Gene Expression Omnibus (GEO) database, CD4^+^ T cells, CD8^+^ T cells, CD274 and immune-related pathways were strongly correlated with markers associated with adipose differentiation (Fig. 1I, J). We next applied CYRBERSORT to analyze the relative levels of distinct immune cell types of NA and CAA tissues. The results indicated that CD8^+^ T cells were significantly decreased, whereas M2 macrophages and neutrophils were increased in CAA compared with NA tissues (Fig. 1K). Based on the immune cell analysis, we established an immunoscore and found that B cells, CD8^+^ T cells and tumor-infiltrating lymphocytes were markedly decreased in CAAs compared with NAs (Fig. 1L). Additionally, we validated adipose differentiation-related proteins and immune-related markers in patient samples. Compared with NA, adipose differentiation-related markers, including FABP4 and adiponectin, were downregulated in CAAs. TCGA data showed that lower expression of adipocyte-related markers, like FABP4, adiponectin, perilipin1, and perilpin5, was associated with worse survival outcomes of breast cancer (Supplementary Fig. S6). Moreover, CAAs were correlated with lower CD4^+^ T and CD8^+^ T cells and higher CD274 expression compared with NA (Fig. 1M, N). Collectively, the above results demonstrated that CAAs exhibited a decreased ability to differentiate and were part of a unique immunosuppressive microenvironment in TNBC.

### CAA promoted TNBC growth, epithelial–mesenchymal transition and metastasis

**Fig. 2:**
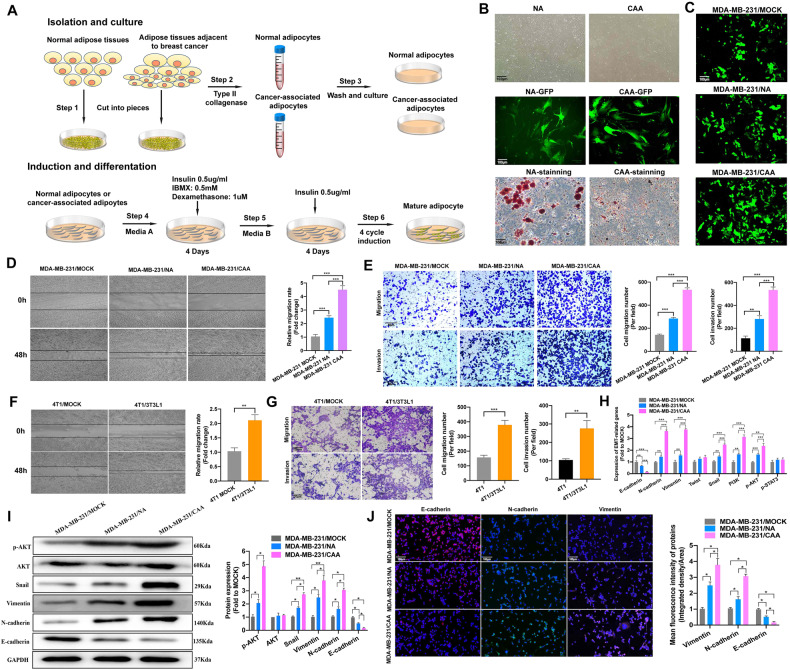
CAA promotes TNBC proliferation, migration, and invasion in vitro. **A** Diagram of the isolation and induced differentiation process of NA and CAA tissues isolated from benign breast neoplasm and TNBC, respectively. **B** NA and CAA morphology before and after induced differentiation. **C** The morphology of MDA-MB-231 cells after coculture with NAs or CAAs for 7 days. **D** Representative images of wound healing assays and statistical analysis of MDA-MB-231 cells after coculture with NAs or CAAs for 7 days. **E** Representative images of transwell assays and statistical analysis of MDA-MB-231 cells after coculture with NAs or CAAs for 7 days. **F** Representative images of wound healing assays and statistical analysis of 4T1 cells after coculture with or without 3T3L1 cells for 7 days. **G** Representative images of transwell assays and statistical analysis of 4T1 cells after coculture with or without 3T3L1 cells for 7 days. **H**–**J** Gene and protein expression of EMT-related markers and the PI3K/AKT and JAK/STAT3 pathways in MDA-MB-231 cells after coculture with NAs or CAAs for 7 days. CAA cancer-associated adipocyte, EMT epithelial–mesenchymal transition, NA normal adipocyte, TNBC triple-negative breast cancer. Data are presented as the means ± SD of at least three independent experiments. *P < 0.05, **P < 0.01, ***P < 0.001.

We successfully cultured NA and CAA cells in vitro (Fig. 2A). No differences in NA and CAA cellular morphology were observed. However, after 28 days of differentiation induction followed by red oil staining, CAAs exhibited a decreased accumulation of lipid droplets compared with NA (Fig. 2B). Considering the crosstalk between TNBC and adipocytes, we then investigated the paracrine effect of CAAs on the responsiveness of TNBC. Thus, we cocultured NA or CAAs with MD-MB-231 cells for 7 days (Fig. 2C). The MDA-MB-231 cell migration rate and invasion abilities were remarkably increased after coculture with CAAs compared with NAs (Fig. 2D, E). Similarly, 4T1 cell migration and invasion abilities were also increased after coculture with 3T3L1 cells (Fig. 2F, G). We also investigated the activated signaling pathways. The results indicated that the PI3K/AKT pathway was stimulated, whereas the JAK/STAT3 pathway was not (Fig. 2H, I). Moreover, we detected the expression of EMT-related markers in MDA-MB-231 cells after coculture with NAs or CAAs. The expression of E-cadherin was decreased, whereas N-cadherin, Vimentin and snail expression were increased after coculture with CAAs (Fig. 2H, J).

**Fig. 3:**
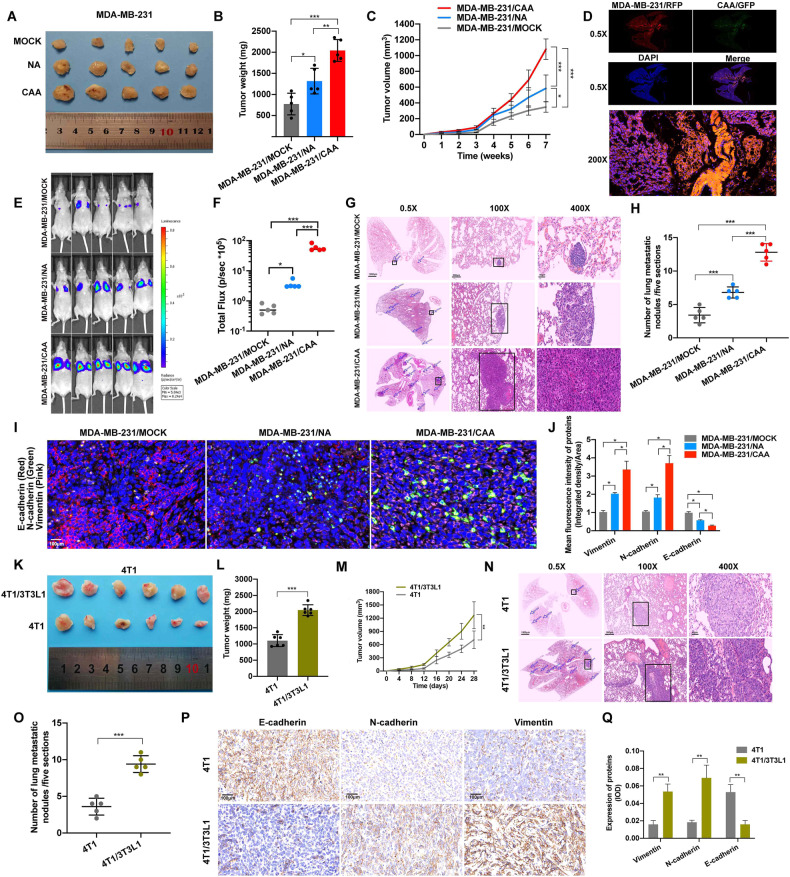
CAA promotes TNBC growth and metastasis in vivo. **A** Images of subcutaneous tumors in nude mice of the MOCK (*n* = 5), NA (*n* = 5) or CAA (*n* = 5) groups. Tumors were obtained after 1.0 × 10^6^ MDA-MB-231 cells per mouse without or with 2.5 × 10^5^ NAs or CAAs were injected into the subcutaneous fat pad of the nude mice for 7 weeks. **B** Tumor weight of the nude mice at the time of sacrifice. **C** A line graph comparing tumor growth of the nude mice. **D** MDA-MB-231 and CAA cells were transfected with RFP and GFP, respectively, and cells were mixed for vein injection. **E**, **F** Luciferase imaging over 2 weeks after injection is shown. Red represents the highest luciferase signal intensity. **G**, **H** Representative images and statistical calculation of tumor metastatic nodules in hematoxylin and eosin (HE)-stained lung sections from nude mice. **I**, **J** Triple fluorescent staining of EMT-related markers and statistical analysis of tumors from nude mice. **K** Images of subcutaneous tumors in BALB/c mice of the 4T1/4T3L1 (*n* = 6) and 4T1 (*n* = 6) groups. Tumors were obtained after 1.0 × 10^6^ 4T1 cells per mouse with or without 2.5 × 10^5^ 3T3L1 cells were injected into the subcutaneous fat pad of the nude mice for 28 days. **L** Tumor weight of the BALB/c mice at the time of sacrifice. **M** A line graph comparing the tumor growth of BALB/c mice. **N**, **O** Representative images and statistical calculation of tumor metastatic nodules in HE-stained lung sections from BALB/c mice. **P**, **Q** IHC staining of EMT-related markers and statistical analysis of tumors from BALB/c mice. CAA cancer-associated adipocyte, EMT epithelial–mesenchymal transition, GFP green fluorescent protein, HE hematoxylin and eosin, IHC immunohistochemistry, NA normal adipocyte, RFP red fluorescent protein, TNBC triple-negative breast cancer. Data are presented as the means ± SD of at least three independent experiments. *P < 0.05, **P < 0.01, ***P < 0.001.

We also investigated whether CAAs could promote tumor progression in vivo. In this study, subcutaneous xenograft models of nude mice were generated. In the subcutaneous model of MDA-MB-231 cells, CAAs promoted TNBC tumor growth (Fig. 3A–C) and lung metastasis (Fig. 3D–H) compared with NAs. IHC of the tumors indicated that CAAs could decrease the expression of E-cadherin, and increase N-cadherin, Vimentin, and snail expressions than NAs in vivo (Fig. 3I, J). In addition, subcutaneous homograft models of BALB/c mice were also generated. In the models, 3T3L1 adipocytes promoted 4T1 cell proliferation (Fig. 3K–M) and lung metastasis (Fig. 3N–O). In addition, it was also demonstrated that 3T3L1 adipocytes also promote EMT of 4T1 cells in vivo (Fig. 3P, Q). Thus, the above experimental data support the conclusion that CAAs promoted TNBC cell growth, EMT and metastasis both in vitro and in vivo.

### CAA-derived CXCL8 promoted TNBC growth and metastasis

**Fig. 4:**
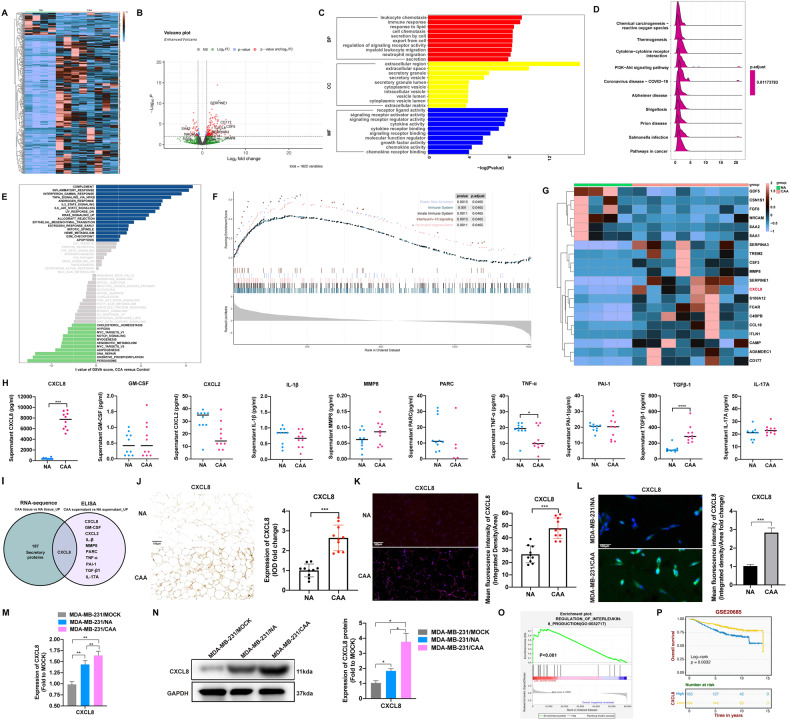
CXCL8 is higher expressed and secreted in CAAs compared with NAs. **A** Heatmap showing the transcriptomic expression levels of the secretory proteins in 10 NA and 10 CAA tissues. **B** Volcano plots depict DEGs of secretory proteins. **C**, **D** GO and KEGG enrichment scatter plot for upregulated transcriptome genes for the secretome in CAAs compared with NAs. **E**, **F** GSVA and GSEA pathway enrichment analysis of upregulated transcriptome genes for the secretome. **G** Heatmap of the top 20 differentially expressed transcriptome genes in the secretome. **H** ELISAs detected 11 secretory proteins in NA and CAA supernatants. **I** Two overlapping sets with Veen diagrams of upregulated secretory proteins between RNA-seq and ELISA. **J**, **K** IHC and IF staining of CXCL8 in 10 NA and 10 CAA tissues. **L** IF staining of NAs and CAAs. **M**, **N** CXCL8 gene and protein expression in MDA-MB-231 cells after coculture without or with NAs or CAAs for 7 days. **O** GSEA of the “INTERLEUKIN-8_PRODUCTION” gene set in the CAAs versus NAs. **P** Overall survival analysis of independent breast cancer cohort GSE20685 for the prognostic potential of CXCL8. CAA cancer-associated adipocyte, CXCL8 C-X-C motif chemokine ligand 8, DEGs differentially expressed genes, ELISA enzyme-linked immunosorbent assay, GSEA gene set enrichment analysis, GSVA gene set variation analysis, IF immunofluorescence, IHC immunohistochemistry, NA normal adipocyte. Data are presented as the means ± SD of at least three independent experiments. *P < 0.05, **P < 0.01, ***P < 0.001.

To identify CAA-specific secreted molecules that lead to progression and EMT, we analyzed the transcriptome levels of secretory proteins. Gene expression profiling of the secretory proteins was performed on NA and CAA tissues (Fig. 4A, B). GO analysis revealed that the upregulated DEGs were associated with the immune response and cellular secretion in the biological process (BP) term, secretory granule, and secretory vesicle in the cellular component (CC) term, and cytokine activity and cytokine binding activity in the molecular function (MF) term. Cytokine‒cytokine receptor interactions were enriched as revealed by KEGG pathway analysis (Fig. 4C, D, Supplementary Fig. S9). The inflammatory response and EMT pathways were enriched via GSVA pathway analysis of upregulated transcriptome genes for the secretory proteins (Fig. 4E). Gene set enrichment analysis (GSEA) indicated that the immune system pathway was enriched (Fig. 4F).

To determine the main secretory proteins that exert their biological role in TNBC, the top 20 secretory proteins were selected for additional consideration (Fig. 4G). We also performed ELISA to detect the concentrations of secretory proteins in NA and CAA supernatants. We found that CXCL8 was expressed at significantly higher levels in CAA supernatant compared with NA supernatant (Fig. 4H, I). The differential expression of CXCL8 between NA and CAA tissues was evaluated. CXCL8 expression was higher in CAAs compared with NA based on IHC (Fig. 4J) and IF (Fig. 4K) analyses. In addition, IF, RT-qPCR and WB confirmed that CAAs exhibit higher CXCL8 expression levels compared with NAs (Fig. 4L–N). Moreover, GSEA of the “INTERLEUKIN-8_PRODUCTION” gene set was enriched in CAA (Fig. 4O). Independent breast cancer cohort GSE20685 dataset showed that higher CXCL8 predicted worse survival (Fig. 4P). Moreover, survival curves of the relapse-free survival in the dataset GSE22219 and GSE58812 cohorts inferred higher CXCL8 expressions also predicted a higher relapse rate (Supplementary Fig. S7).

**Fig. 5:**
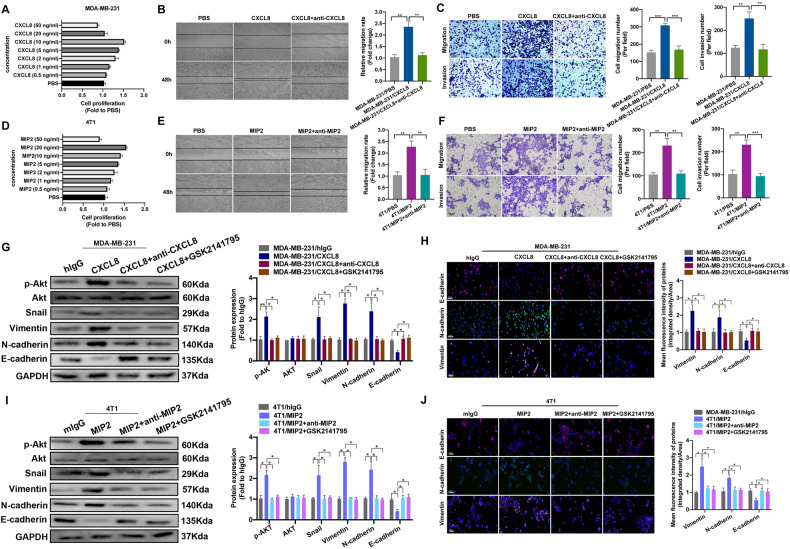
CXCL8 aggravates TNBC proliferation, migration, invasion, and EMT via the PI3K/AKT pathway. **A** Viability of MDA-MB-231 cells treated with different concentrations of CXCL8. **B**, **C** Representative images of wound healing and Transwell assays of MDA-MB-231 cells after treatment with CXCL8 (10 ng/ml) or CXCL8 (10 ng/ml) + CXCL8 antibody (10 μg/ml) for 7 days. **D** Viability of 4T1 cells treated with different concentrations of MIP2. **E**, **F** Representative images of wound healing and transwell assays of 4T1 cells after treatment with MIP2 (20 ng/ml) or CXCL8 (20 ng/ml) + MIP2 antibody (10 μg/ml) for 7 days. **G**, **H** Western blot and IF analysis of the EMT-related markers AKT and p-AKT in MDA-MB-231 cells after treatment with CXCL8 (10 ng/ml) or CXCL8 (10 ng/ml) + CXCL8 antibody (10 μg/ml) or CXCL8 (10 ng/ml) + GSK2141795 (10 μmol/L) for 7 days. **I**, **J** Western blot and IF analysis of the EMT-related markers AKT and p-AKT in MDA-MB-231 cells after treatment with MIP2 (20 ng/ml) or MIP2 (20 ng/ml) + MIP2 antibody (20 μg/ml) or MIP2 (10 ng/ml) + GSK2141795 (10 μmol/L) for 7 days. CXCL8 C-X-C motif chemokine ligand 8, DEGs differentially expressed genes, ELISA enzyme-linked immunosorbent assay, EMT epithelial–mesenchymal transition, GSEA gene set enrichment analysis, GSVA gene set variation analysis, IF immunofluorescence, IHC immunohistochemistry, MIP2 macrophage inflammatory protein-2, TNBC triple-negative breast cancer. Data are presented as the means ± SD of at least three independent experiments. *P < 0.05, **P < 0.01, ***P < 0.001.

To investigate the function of CXCL8 in the progression and metastasis of TNBC, we performed in vitro experiments. Cell viability assays indicated that 10 ng/ml CXCL8 exhibited the strongest ability to promote proliferation (Fig. 5A, Supplementary Fig. S8). Furthermore, we next assessed the effects of CXLC8 and the potential inhibitory activity of the monoclonal anti-CXCL8 antibody on cell biological function. CXCL8 promotes MDA-MB-231 cell migration and invasion, whereas blocking CXCL8 with a specific antibody reverses this effect (Fig. 5B, C). Assessment of the role of CXCL8 in the immune microenvironment using murine models has been hampered by the fact that CXCL8 has not been detected in rodents; however, previous studies indicated that macrophage inflammatory protein 2 (MIP-2) in mice could represent a surrogate of CXCL8 in humans [41, 42]. We also assessed the effect of different concentrations of MIP2 on 4T1 cell proliferation, and 20 ng/ml was the optimal concentration to promote cell proliferation (Fig. 5D, Supplementary Fig. S8). Next, we analyzed the effects of MIP2 and the monoclonal anti-MIP2 antibody on 4T1 progression. As expected, MIP2 facilitated 4T1 cell migration and invasion, whereas blocking MIP2 using an anti-MIP2 antibody had direct inhibitory effects on tumor progression (Fig. 5E, F). The role of CXCL8 in regulating EMT was also assessed. As expected, CXCL8 increased Vimentin and N‐cadherin expression but decreased E‐cadherin expression, a classic feature of EMT, as shown by WB and IF assays. In addition, the application of the CXCL8 antibody or the AKT inhibitor GSK2141795 reversed the EMT process (Fig. 5G, H). The effect of MIP2 on promoting EMT in 4T1 cells was also verified in vitro (Fig. 5I, J). Therefore, the above results suggested that CXCL8 promotes the TNBC EMT process via the PI3K/AKT pathway.

### CAA-derived CXCL8 modulated CD274 expression and immune cell infiltration

**Fig. 6:**
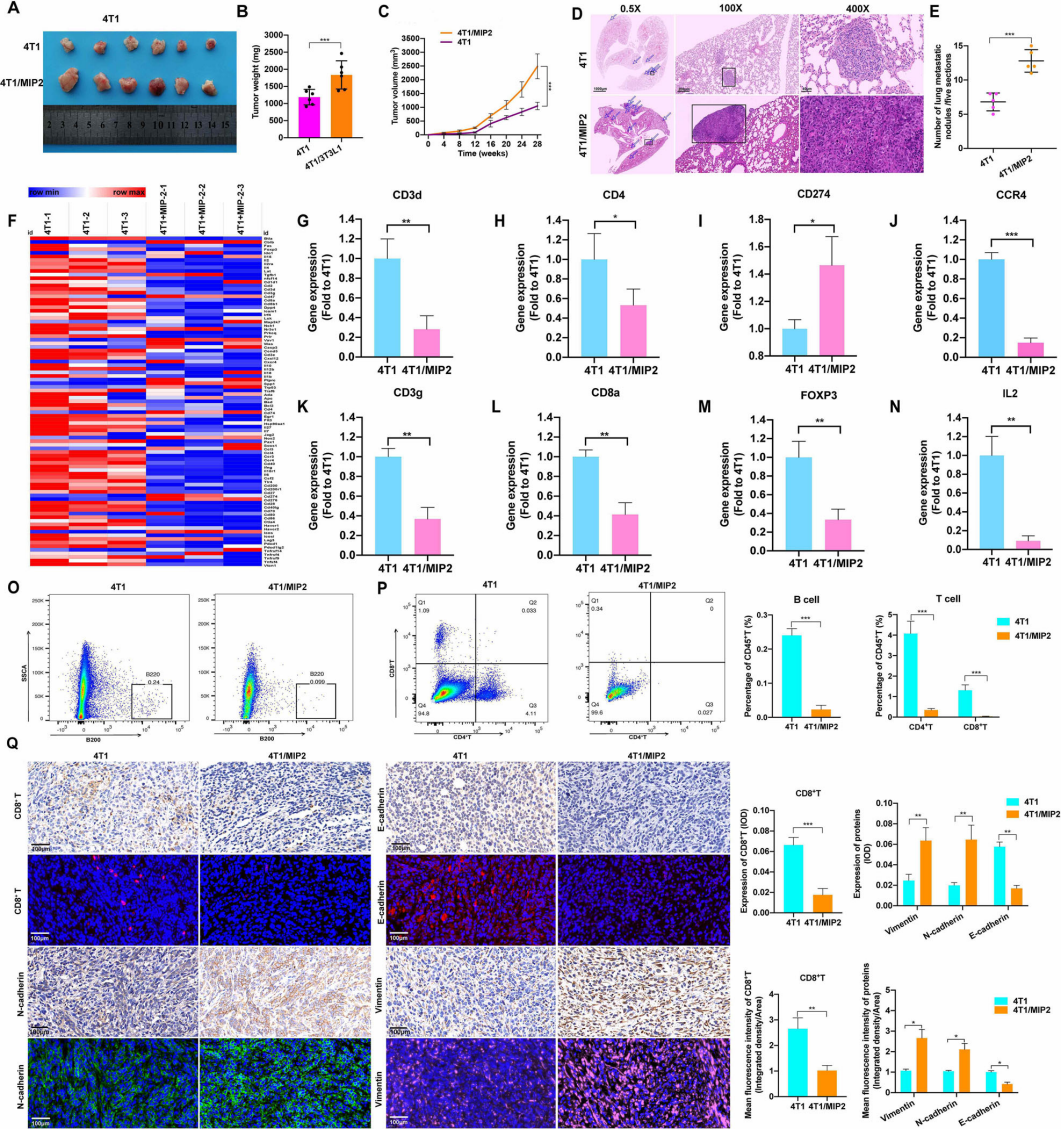
MIP2 promotes TNBC growth and metastasis by upregulating CD274 expression and inhibiting T-cell infiltration. **A** Images of subcutaneous tumors in BALB/c mice in the 4T1 group (*n* = 6) and the 4T1/MIP2 group (4T1 cells treated with MIP2 (20 ng/ml) for 7 days) (*n* = 6). **B** Tumor weight of the BALB/c mice at the time of sacrifice. **C** A line graph comparing the tumor growth of BALB/c mice. **D**, **E** Representative images and statistical calculation of tumor metastatic nodules in HE-stained lung sections from BALB/c mice. **F** Heatmap of the immune-related gene transcriptomic signature of tumors in the 4T1 and 4T1/MIP2 groups. **G**–**N** The expression levels of CD3d, CD4, CD274, CCD4, CD3g, CD8a, FOXP3 and IL2 in the 4T1 and 4T1/MIP2 groups. **O**, **P** Flow cytometry analysis of T cells, B cells in the 4T1 and 4T1/MIP2 groups. **Q** Representative images of IHC and IF staining and their statistical analysis of EMT-related markers and CD8^+^ T cells in the 4T1 and 4T1/MIP2 groups. EMT epithelial–mesenchymal transition, HE hematoxylin and eosin, IF immunofluorescence, IHC immunohistochemistry, MIP2 macrophage inflammatory protein-2, CD274 programmed death-ligand 1, TNBC triple-negative breast cancer. Data are presented as the means ± SD of at least three independent experiments. *P < 0.05, **P < 0.01, ***P < 0.001.

Next, we investigated the effect of CXCL8 on immunity. In our study, CXCL2/MIP-2 in mice was employed as a surrogate for human CXCL8 because the human CXCL8 gene has not been detected in rodents. We successfully established a mouse model in BALB/c mice using 4T1 cells cocultured with or without MIP2. In contrast with 4T1 cells, 4T1 cells cocultured with MIP2 cells exhibited dramatically increased subcutaneous tumor growth and lung metastasis in the subcutaneous tumor model as demonstrated by an increased tumor weight, tumor volume and lung involvement (Fig. 6A–E). To explore whether CXCL8 exerts an important role in the immune response, we conducted PCR array analysis to identify MIP2-stimulated immune-related genes (Fig. 6F). Further analysis found that MIP2 stimulated the expression of CD3d, CD4, CCR4, CD3g, CD8a, FOXP3, and IL2 while inhibiting the expression of CD274, indicating that MIP2 played an immunosuppressive role (Fig. 6G–N). Consequently, flow cytometry further demonstrated that MIP2 inhibited the immune response by decreasing B- and T-cell infiltration while increasing macrophage and neutrophil infiltration (Fig. 6O, P). IHC and IF assays also indicated that MIP2 contributed to a decrease in CD8^+^ T-cell infiltration and to the promotion of EMT in vivo (Fig. 6Q). All the above evidence indicates that CXCL8 secreted from CAA plays an immunosuppressive role by elevating CD274 levels and inhibiting immune cell infiltration (Fig. 6F–R, Supplementary Fig. S10).

### Targeting CAA-derived CXCL8 sensitized TNBC to anti-PD-1 immunotherapy by reversing the immunosuppressive microenvironment

**Fig. 7:**
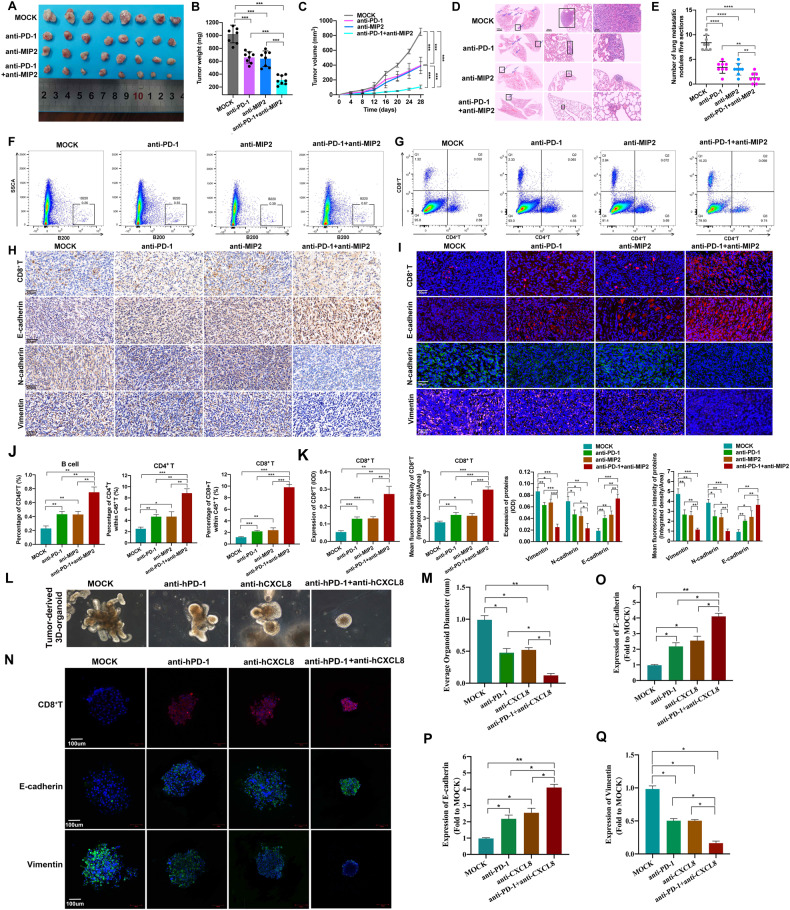
Combined blockade of PD-1 and MIP2 modulates immune infiltration and results in synergistic antitumor efficacy in TNBC. **A** Photo of excised subcutaneous tumors of BALB/c mice from different treatment groups. The injection of the anti-PD-1 antibody (10 μg once) and/or anti-MIP2 antibody (10 μg once) was scheduled every 2 days. **B**, **C** Tumor weight and growth curves of mice with different treatments. **D**, **E** Representative images and statistical calculation of tumor metastatic nodules in HE-stained lung sections from BALB/c mice. **F**, **G**, **J** Flow cytometry analysis of T and B cells in different treatment groups. **H**, **I**, **K** Representative images of IHC and IF staining and statistical analysis of EMT-related markers and CD8^+^ T cells in different treatment groups. **L** tumor tissues of a TNBC patient were used to form 3D-organoids in culture. Images are shown of organoids after treated with hCXCL8 or/and hPD-1. **M** Quantification of the average organoid diameter of different groups. **N**–**Q** IF and statistical analysis of CD8^+^T, E-cadherin, and vimentin in different treatment groups of PDO. EMT epithelial–mesenchymal transition, HE hematoxylin and eosin, IF immunofluorescence, IHC immunohistochemistry, MIP2 macrophage inflammatory protein-2, PD-1 programmed cell death protein 1, TNBC triple-negative breast cancer, hCXCL8 human C-X-C Motif Chemokine Ligand 8, hPD-1 human programmed cell death protein 1, PDO patient-derived organoid. Data are presented as the means ± SD of at least three independent experiments. *P < 0.05, **P < 0.01, ***P < 0.001.

Currently, we demonstrated that CAA-derived CXCL8 can modulate the immunosuppressive microenvironment. Therefore, we systemically evaluated the combination of targeting the CXCL8 pathway and PD-1 by administering antibodies. CXCL8 has not been detected in rodents, murine chemokines, CXCL1/KC or CXCL2/MIP2 is suitable for the compensate for lack of CXCL8. We both tried the effect of CXCL1/KC or CXCL2/MIP2 in vivo models. However, combination blockade of PD-1 and CXCL1/KC did not achieve better synergistic antitumor efficacy like CXCL2/MIP2 (Fig. 7A–C, Supplementary Fig. S11A–C). Thus, we established a BALB/c mouse model bearing 4T1 cells treated with MIP2 to analyze the role of CXCL8 in the immune microenvironment. Then, tumor mouse models bearing 4T1 cells were randomized into groups treated with either MIP2 antibody or/and anti-PD-1 antibody. Consequently, monotherapy with either MIP2 antibody or anti-PD-1 antibody exhibits fair inhibition of tumor growth compared with no therapy. Moreover, tumor growth was also markedly decreased in response to treatment with the combination therapy compared with monotherapy. Additionally, we also detected lung metastasis nodules in each group. It was observed that lung metastasis could be mitigated by targeting MIP2 or PD-1. Combination therapy led to significant decreases in lung metastasis compared with monotherapy (Fig. 7D, E). In addition, targeting either MIP2 or PD-1 inhibited the EMT process. Moreover, in contrast with monotherapy, the combination therapy had a stronger capacity to inhibit EMT (Fig. 7H–K). Also, combined blockade of PD-1 and MIP2 synergistically reduced cell proliferation, and angiogenesis, decreased cell stemness, and induced cell apoptosis (Supplementary Fig. S12). The antitumor immunity activity was determined by blocking MIP2 and/or PD-1. We assessed the levels of CD4^+^ T cells, CD8^+^ T cells, macrophages, and neutrophils with flow cytometry in the tumor tissues of the treated animals. As expected, treatment with MIP2 antibody and/or anti-PD-1 was positively associated with the infiltration of CD4^+^ T cells and CD8^+^ T cells but negatively correlated with macrophages and neutrophils (Fig. 7F–K, Supplementary Figs. S13 and S14). To conclude, blocking the MIP2 pathway and PD-1/CD274 jointly stimulated the antitumor immune response.

To further support our conclusions, our team established a TNBC patient’s tumor-derived 3D organoid (Fig. 7L). Then, the patient-derived organoid (PDO) model was randomized into groups treated with either human CXCL8 (hCXCL8) antibody or/and human PD-1 (hPD-1) antibody. Consequently, monotherapy with either hCXCL8 antibody or hPD-1 antibody exhibits fair inhibition of tumor growth compared with no therapy based on the morphology of PDO. In addition, we also detect the expressions of CD8^+^ T, E-cadherin, and vimentin. Results have shown that treatment with hCXCL8 antibody and/or hPD-1 was positively associated with the infiltration of CD8^+^ T cells. And, blocking hCXCL8 and the hPD-1 pathway acts synergistically to inhibit TNBC growth and EMT process (Fig. 7N–Q). Thus, the combination of targeting CXCL8 and blocking the PD-1 pathway synergistically increased the tumor immune response and inhibited tumor progression.

**Fig. 8:**
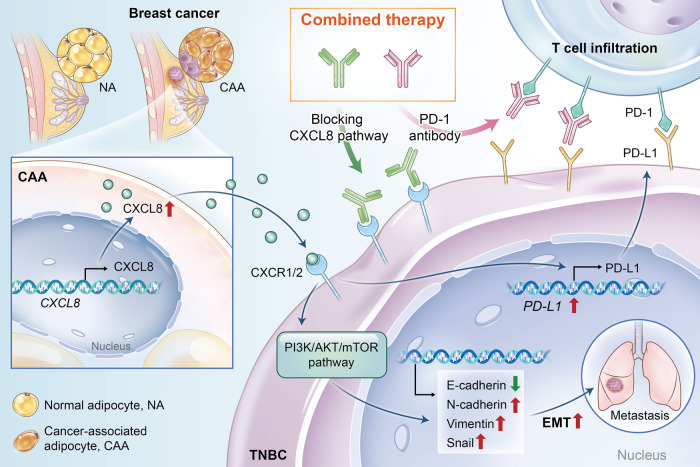
Working model. In TNBC, CAA is positively associated with an immunosuppressive microenvironment. CAA is highly expressed and secretes CXCL8, which promotes tumor growth and lung metastasis and regulates EMT through the PI3K/AKT pathway. CXCL8 modulated the immunosuppressive microenvironment by upregulating CD274 expression and suppressing T-cell infiltration. Blockade of the CAA-derived CXCL8 pathway can sensitize the immunotherapy response of PD-1, thus inhibiting TNBC progression. CAA cancer-associated adipocyte, NA normal adipocyte, PD-1 programmed cell death protein 1, CD274 programmed death-ligand 1.

All the above results demonstrate that the combination of targeting CAA-derived CXCL8 and anti-PD-1 could result in a synergistic antitumor effect in TNBC by reversing the immunosuppressive microenvironment. We provided a schematic to unravel the complex theory that targeting CAA-derived CXCL8 enhances the efficacy of anti-PD-1 immunotherapy and improves TNBC outcomes. CXCL8, a secretory protein highly expressed in CAAs, promoted TNBC cell progression and lung metastasis and induced the EMT process via the PI3K/AKT pathway. CXCL8 forged a highly immunosuppressive ecosystem by increasing CD274 expression and decreasing T- and B-cell infiltration (Fig. 8).

## Discussion

Accounting for approximately 15–20% of breast cancers, TNBC is a biologically and clinically heterogeneous malignant disease [11]. TNBC still remains a huge challenge in clinical treatment due to its strong invasiveness, high incidence of distant metastasis and lack of efficiency and well-organized therapeutic targets, all of which are associated with the poor prognosis of TNBC [43]. The abundant immune cell infiltration of TNBC has provided a huge opportunity for immunotherapy with Immune Checkpoint Inhibitors (ICIs) [11]. Moreover, clinical trials have demonstrated that advanced TNBC patients with more infiltration of TILs are more likely to benefit from ICIs [44, 45]. Although ICIs could improve survival outcomes, a proportion of TNBC patients do not achieve obvious clinical benefits from immunotherapy. Therefore, how to enhance the efficacy of ICIs in TNBC still needs to be investigated.

Adipocytes are the primary cellular components with the largest population that comprise the TME. Emerging evidence suggests that adipocytes participate in intimate mutual and dynamic crosstalk with cancer cells and promote cancer cell migration, invasion and metastasis and drug resistance [25, 46]. Influenced by cancer cells, NAs are driven to become CAAs, which are characterized by a fibroblast-like phenotype, reduced differentiation capacity, and the presence of small fat droplets [47]. In this process of transformation, CAAs gradually lose their differentiation capacity through the downregulation of markers associated with adipose differentiation, such as FABP4 and adiponectin [25]. Furthermore, consistent with previous studies, CAA exhibited increased invasiveness and metastatic potential. In the present study, our data implied that CAAs exhibited a decreased differentiation capacity accompanied by a reduction in adipocyte-related markers, including FABP4 and adiponectin, compared with NAs, and it was revealed that CAAs promoted TNBC progression and EMT via the PI3K/AKT pathway.

A major finding of the study is that adipocytes are closely related to the immune response. Transcriptome data analyses showed that the cytokine-mediated signaling pathway and immune response were mainly enriched in CAAs. In this study, we focused on the association of adipocytes with immune infiltration, and CAAs are associated with reduced CD8^+^ T-cell infiltration, higher macrophage and neutrophil levels, and increased CD274 expression compared with NAs. The tumor and surrounding TME have been demonstrated to be in a chronic state of inflammation, and CAAs might profoundly influence the effector functions of immune cells [48, 49]. Additionally, CAAs exert a critical role in the regulation of T cells. CAA-derived leptin influences the metabolic pattern of CD8^+^ T cells through STAT3 activation and fatty acid oxidation, thus leading to the inhibition of CD8^+^ T effector cell function [50]. In our study, we found that CAA was associated with higher CD274 expression and lower CD8^+^ T-cell infiltration in TNBC. In addition, metabolic disorders of CAAs might be associated with immunoregulation and facilitate immune escape.

However, the molecular mechanisms of CAAs in the regulation of the immune response in TNBC remain uncertain. In the present study, we identified CAA-derived CXCL8 as a major secretory cytokine modulating the immunosuppressive microenvironment. CXCL8, a proinflammatory chemokine and a chemoattractant for myeloid leukocytes, is generally considered to be a protumorigenic factor and is associated with poor prognosis in cancer patients [51]. Elevated serum IL-8 levels are associated with poor outcomes in advanced cancer patients who have been treated with ICIs, such as nivolumab and/or ipilimumab [52]. Our in vitro and in vivo experiments showed that CAA-derived CXCL8 promoted TNBC cell proliferation, migration, invasion, lung metastasis and EMT via the PI3K/AKT pathway. The human CXCL8 gene has not been detected in rodents, and MIP-2 in mice is believed to be a homolog of human CXCL8 [41, 42]. Thus, we established a BALB/c mouse model bearing 4T1 cells treated with MIP2 to analyze the role of CXCL8 in the immune microenvironment. The results suggested that CAA-derived CXCL8 suppressed T-cell activation by upregulating CD274 expression. Notably, many studies together with ours have consistently shown that CXCL8 is an angiogenic polypeptide that is expressed in multiple cancers, and it is a common secretory protein that potentially mediates immune reactions in the immune system [53, 54]. In gastric cancer (GC), CXCL8 secreted by macrophages contributes to the immunosuppressive ecosystem by inducing CD274 macrophages. CXCL8 derived from GC mesenchymal stem cells (MSCs) can also induce CD274 expression by targeting c-myc via the STAT3 and mTOR signaling pathways [55]. CXCL8 has been reported to upregulate PD-1 expression in CD8^+^ T cells, thus resulting in an immunosuppressive microenvironment in GC primary tumors and metastatic lymph nodes [56]. IMvigor210 and IMmotion150 clinical trials also found that CXCL8 expression was associated with higher levels of neutrophils in tumors [57]. Additionally, our study further confirmed that higher CXCL8 expression was associated with reduced T-cell infiltration and increased macrophage and neutrophil infiltration, thus creating an immunosuppressive environment. Hence, CXCL8 is representative of a suppressive role that affects the efficiency and clinical benefit of PD-1/CD274 checkpoint blockade therapy. Therefore, our study explains one of the mechanisms by which CAA-derived CXCL8 exhibits an immunosuppressive role and why targeting CXCL8 sensitizes the efficiency of immunotherapy in TNBC. This finding has rarely been reported.

Now that we have demonstrated that CXCL8 is one of the obstacles to the efficacy of immunotherapy, we hypothesized that targeting CXCL8 combined with PD-1 antibody might achieve a synergistic effect against TNBC. Consequently, after combination therapy, the immune microenvironment of TNBC was remodeled, and CD8^+^ T and CD4^+^ T cells were stimulated, thereby making “cold” tumors turn into “hot” tumors with robust T-cell infiltration [58]. Blocking CXCL8 and the PD-1 pathway acts synergistically to not only inhibit TNBC growth and metastasis but also to reverse the immunosuppressive microenvironment, thus enhancing the efficiency of anti-PD-1 immunotherapy and representing a novel therapeutic strategy for TNBC.

In conclusion, the present study describes the characteristics of the cancer-promoting role and systemic immune suppression status of CAA. CAA-derived CXCL8 promoted TNBC growth and metastasis and modulated the immunosuppressive microenvironment by upregulating CD274 expression and suppressing T-cell infiltration. Moreover, the present research reveals a novel therapeutic strategy of targeting CAA-derived CXCL8 to sensitize TNBC to immunotherapy.

### Supplementary information

Supplemental figures and tables

Supplemental file_Uncropped WB

Checklist

## Data Availability

All data generated in the current study are available.
