# Supplementary material for: Targeting cancer-associated adipocyte-derived CXCL8 inhibits triple-negative breast cancer progression and enhances the efficacy of anti-PD-1 immunotherapy
Source: Cell Death Dis. 2023 Oct 28;14(10):703. doi: 10.1038/s41419-023-06230-z (PMC10613226; doi:10.1038/s41419-023-06230-z)
Supplement: Supplementary file 1 — Supplemental figures and tables [file 41419_2023_6230_MOESM1_ESM.pdf]

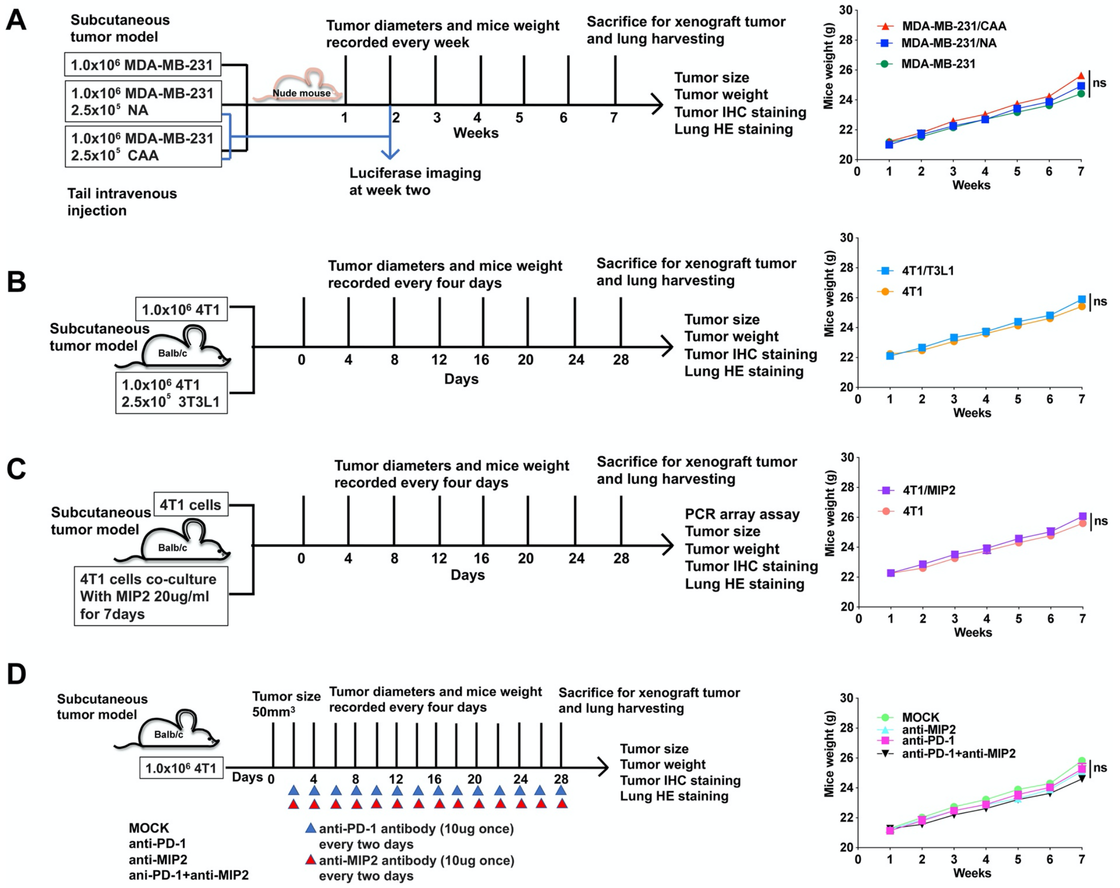

Fig.S1 Schematic diagrams of mice models. A A suspension containing 1.0×10^{6} MDA-MB-231 cells per mouse with or without 2.5×10^{5} NAs or CAAs was injected subcutaneously into nude mice. Luciferase imaging was performed at week two. **B** A suspension mix with 1.0×10^{6} 4T1 cells per mouse with or without 2.5×10^{5} 3T3L1 cells was implanted to establish subcutaneous tumors in female BALB/c mice. **C** 4T1 cells co-cultured with or without MIP2 (20 ng/ml) for 7 days and then cells (1.0×10^{6} cells/mouse) were subcutaneously implanted into female BALB/c mice. **D** 4T1 cells (1.0×106 cells/mouse) were subcutaneously implanted into BALB/c mice, and the anti-PD-1 antibody (10 μg once) and the anti-MIP2 antibody (10 μg once) were injected every two days after the tumor size reached 50 mm^3. And, mice weights of different groups in each mouse model were compared. The tumor diameters were recorded in nude mice every week, while in Balb/c mice every four days. The tumor weight of each mouse was measured at the time of sacrifice. Lung tissue and tumor tissue were scheduled for HE and IHC analysis, respectively. HE: hematoxylin and eosin; IHC: immunohistochemistry; MIP2: macrophage inflammatory protein-2.

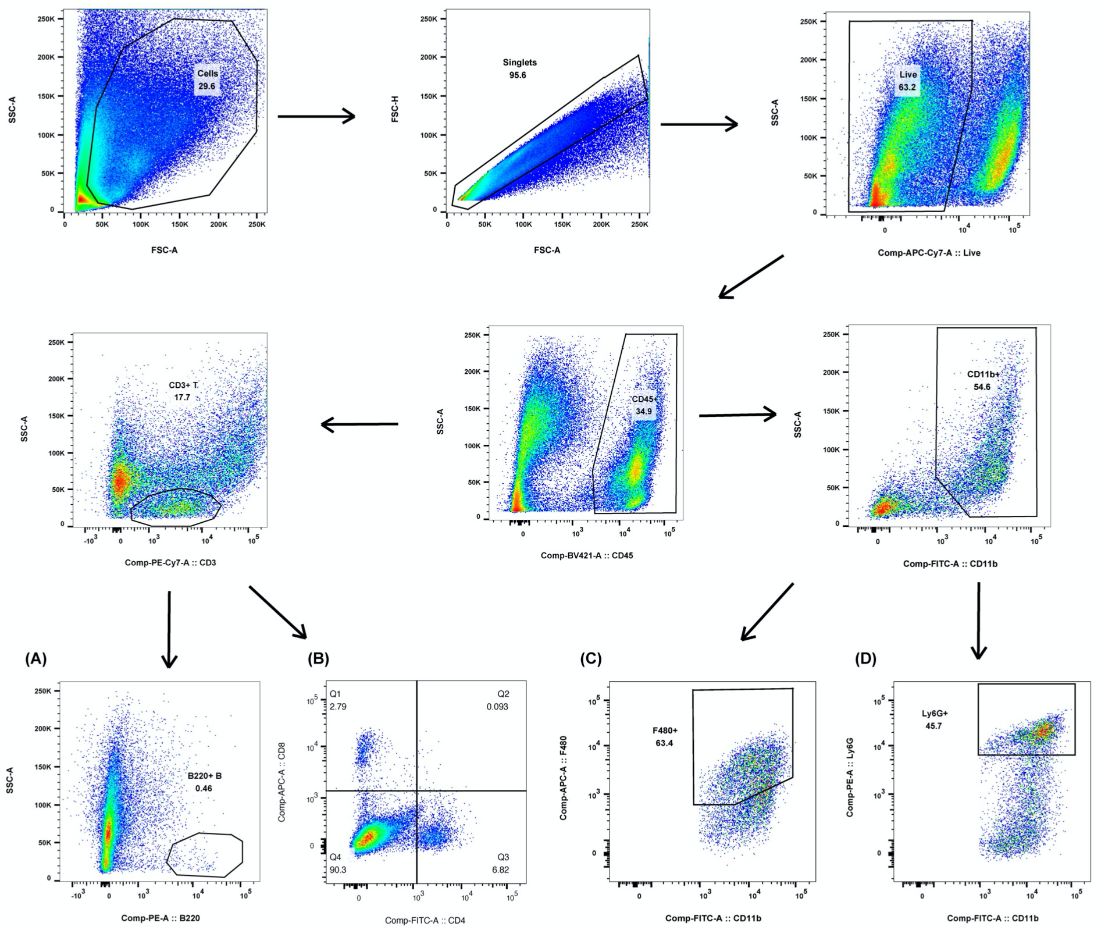

Fig.S2 Flow cytometry gating strategy for identification of immune cells. A B cell subset was identified as CD45$^+$ CD3$^+$ B220$^+$. **B** CD4$^+$ T and CD8$^+$ T cells were identified as CD45$^+$ CD3$^+$ CD4$^+$ or CD8$^+$. **C-D** Macrophage was identified as CD45$^+$ CD11b$^+$ F4/80$^+$, while neutrophile was identified as CD45$^+$ CD11b$^+$ Ly6G$^+$.

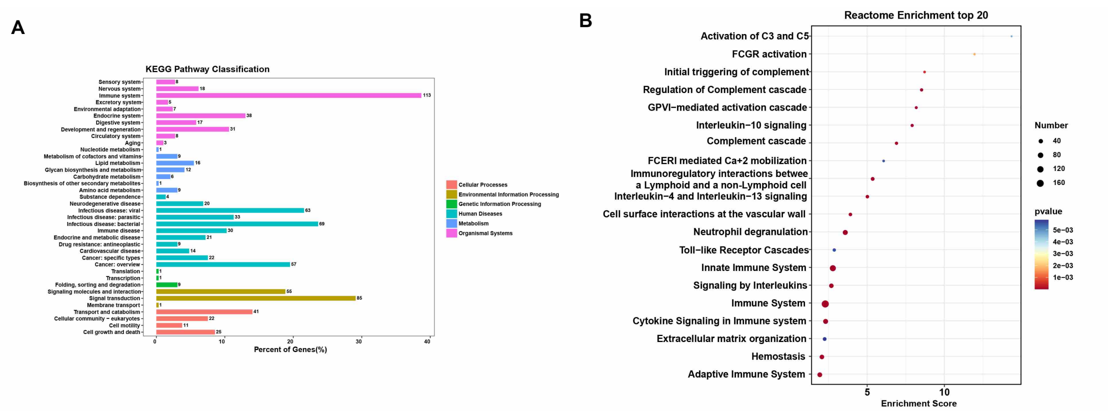

Fig.S3. **A** KEGG pathway analysis of the DEGs in CAAs compared with NAs. **B** The top 20 enriched REACTOME pathways for the upregulated genes in CAAs compared with NAs.

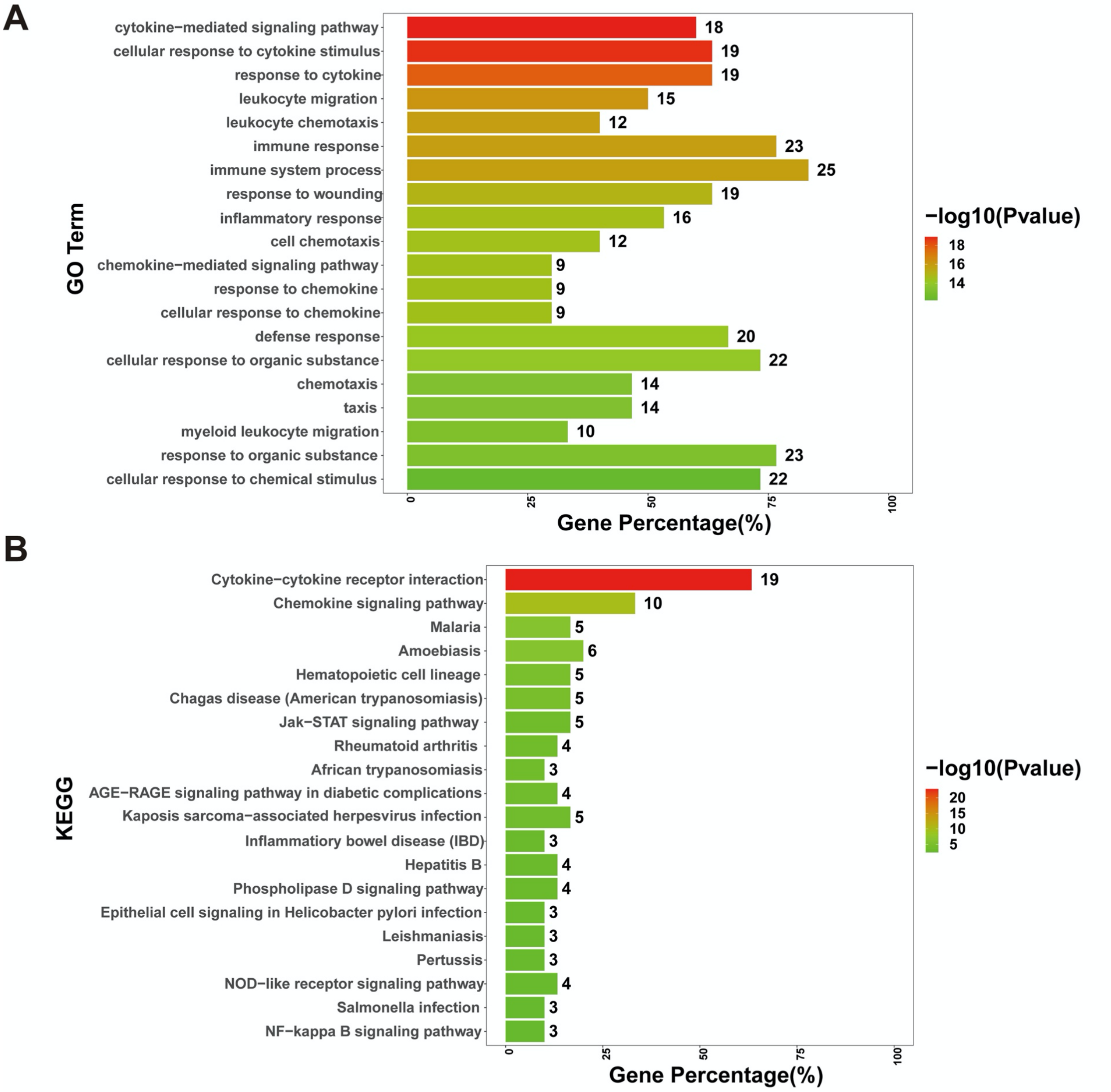

Fig. S4 GO and KEGG enrichment scatter plot for the top 30 upregulated genes in CAAs compared with NAs. GO: Gene Ontology; KEGG: Kyoto Encyclopedia of Genes and Genomes; CAA: cancer-associated adipocyte; NA: normal adipocyte.

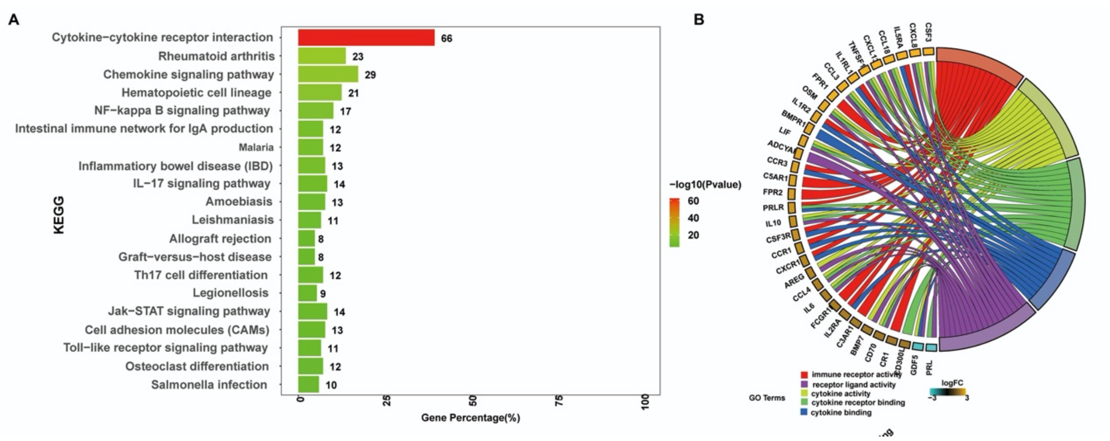

Fig. S5 GO and KEGG enrichment scatter plot for the DEGs of the immune signature genes in CAAs compared with NAs. GO: Gene Ontology; KEGG: Kyoto Encyclopedia of Genes and Genomes; DEGs: differentially expressed genes; CAA: cancer-associated adipocyte; NA: normal adipocyte.

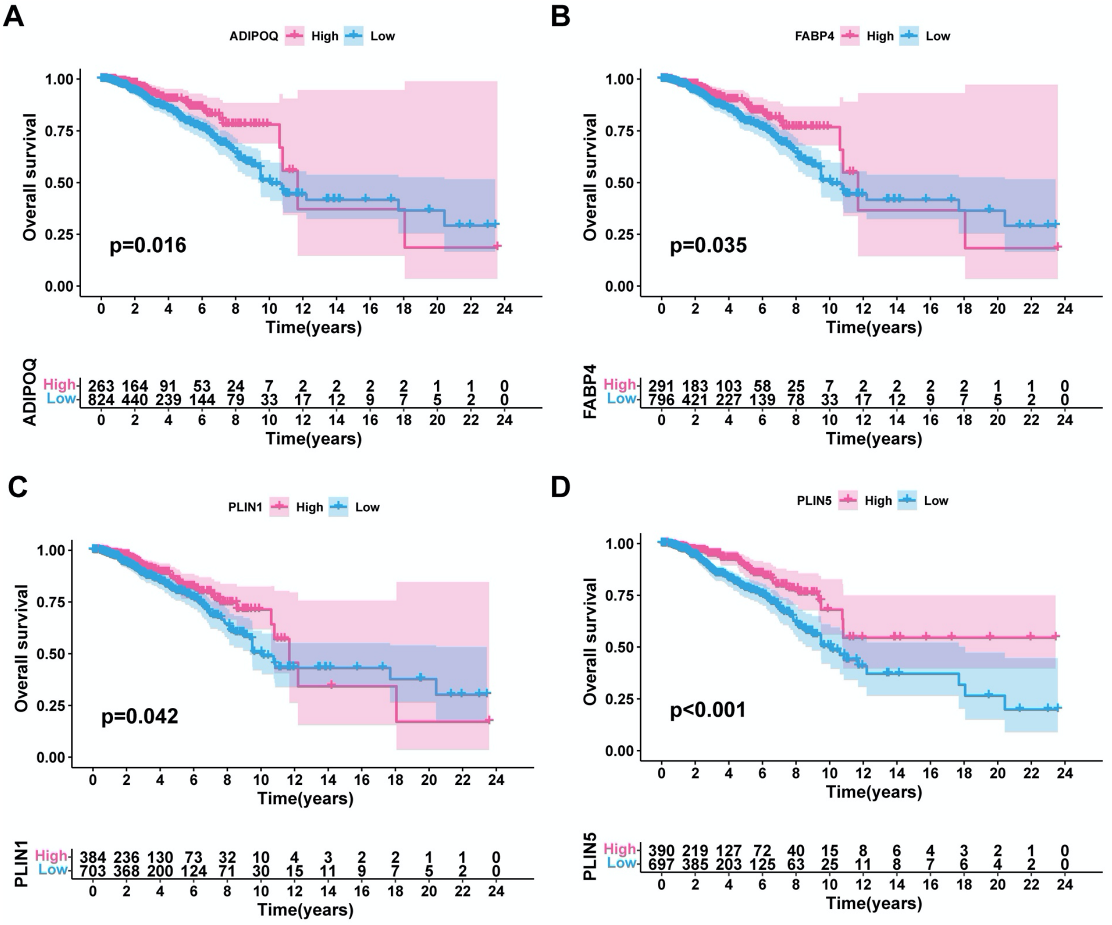

Fig. S6 Impact of adipocyte-related markers on survival outcomes of breast cancer patients. Overall survival analysis of TCGA breast cancer samples for the prognostic potential of adipocyte-related markers, including ADIPOQ, FABP4, PLIN1, and PLIN5. ADIPOQ: adiponectin; FABP4: fatty acid-binding protein 4; PLIN1: perilipin1; PLIN5: perilipin5; TCGA: The Cancer Genome

Atlas.

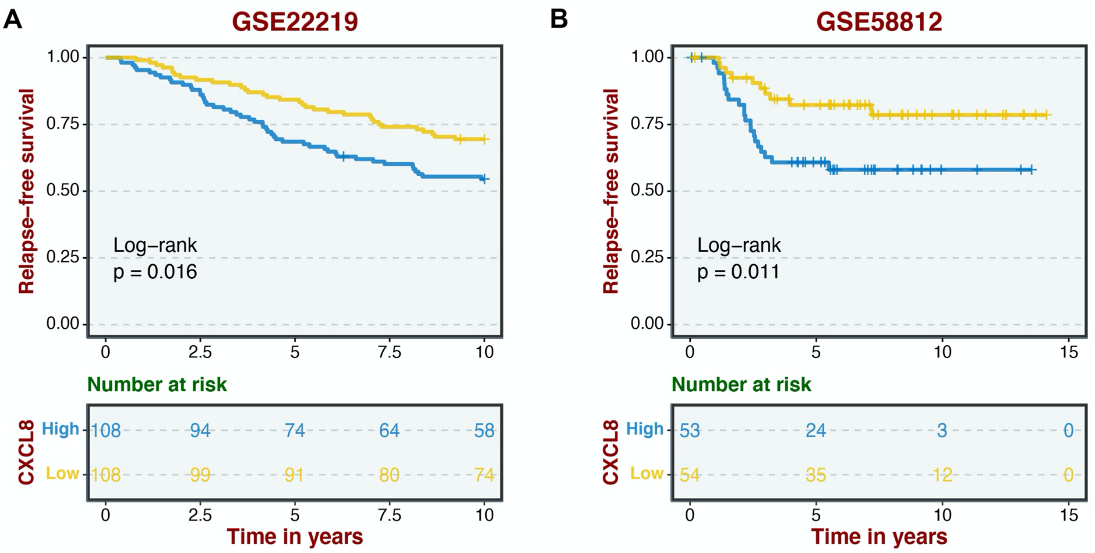

Fig. S7 Kaplan–Meier survival curves of the relapse-free survival in the external dataset GSE22219 and GSE58812 cohorts.

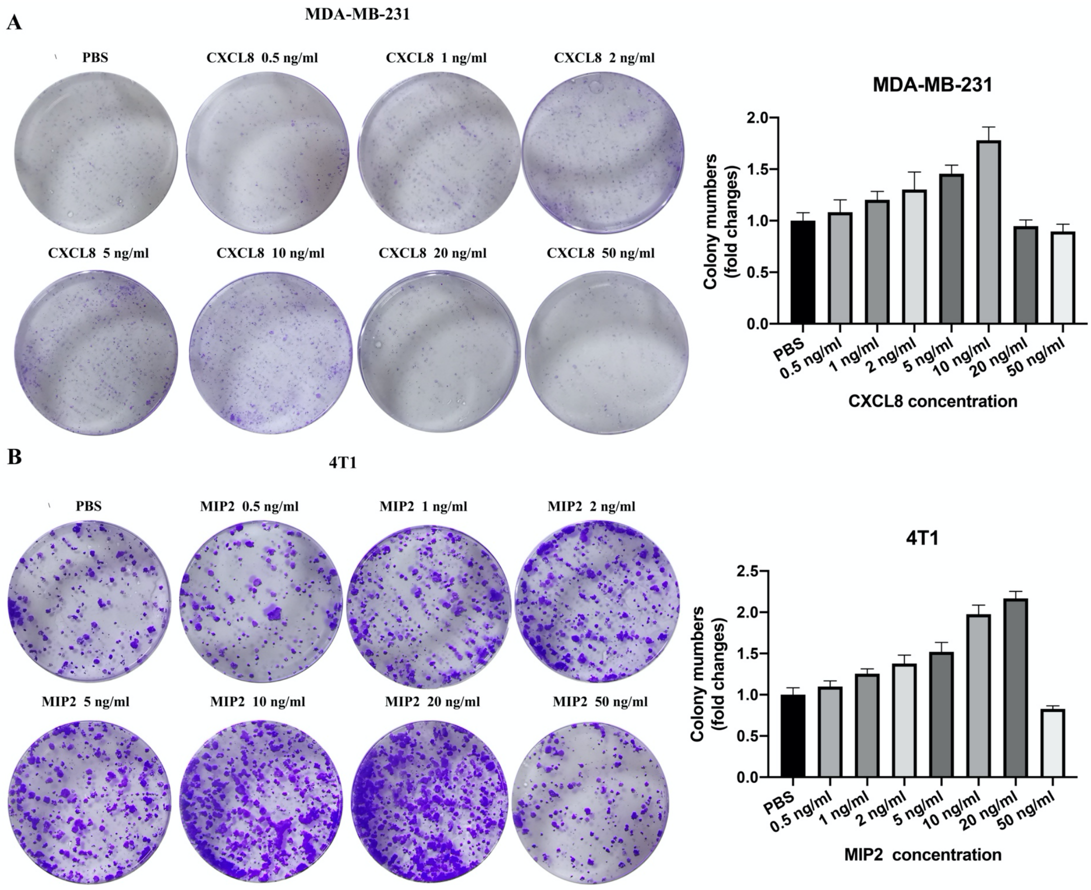

Fig. S8 The cell colony assays of MDA-MB-231 and 4T1 treated with CXCL8 or MIP2. A Cell colony of MDA-MB-231 cells treated with different concentrations (0 ng/ml, 0.5 ng/ml, 1 ng/ml, 2 ng/ml, 5 ng/ml, 10 ng/ml, 20 ng/ml, and 50 ng/ml) of CXCL8. **B** Cell colony of 4T1 cells treated with different concentrations (0 ng/ml, 0.5 ng/ml, 1 ng/ml, 2 ng/ml, 5 ng/ml, 10 ng/ml, 20 ng/ml, and 50 ng/ml) of MIP2. CXCL8: C-X-C motif chemokine ligand 8; MIP2: macrophage inflammatory protein-2.

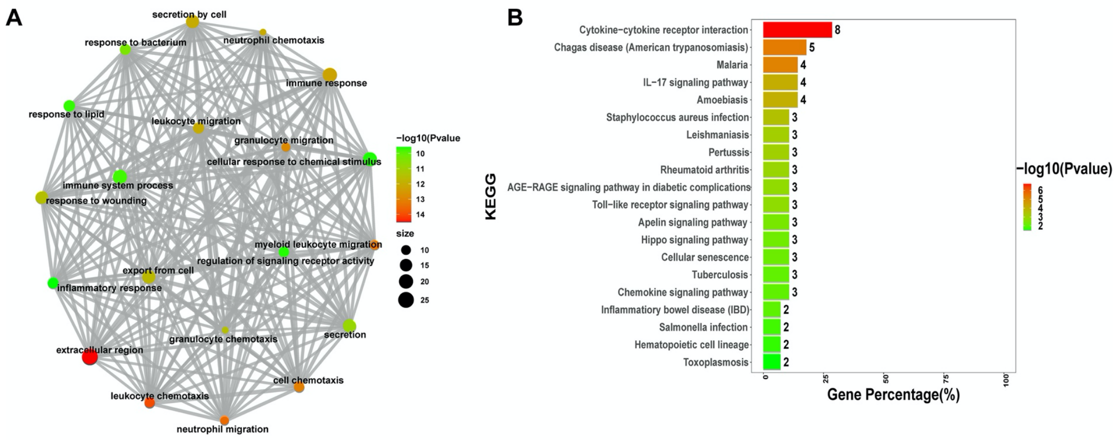

Fig. S9 Gene enrichment of top 30 secretory proteins from CAA. A-B GO and KEGG enrichment scatter plot for the top30 upregulated transcriptome genes for the secretome. CAA: cancer-associated adipocyte; GO: Gene Ontology; KEGG: Kyoto Encyclopedia of Genes and Genomes.

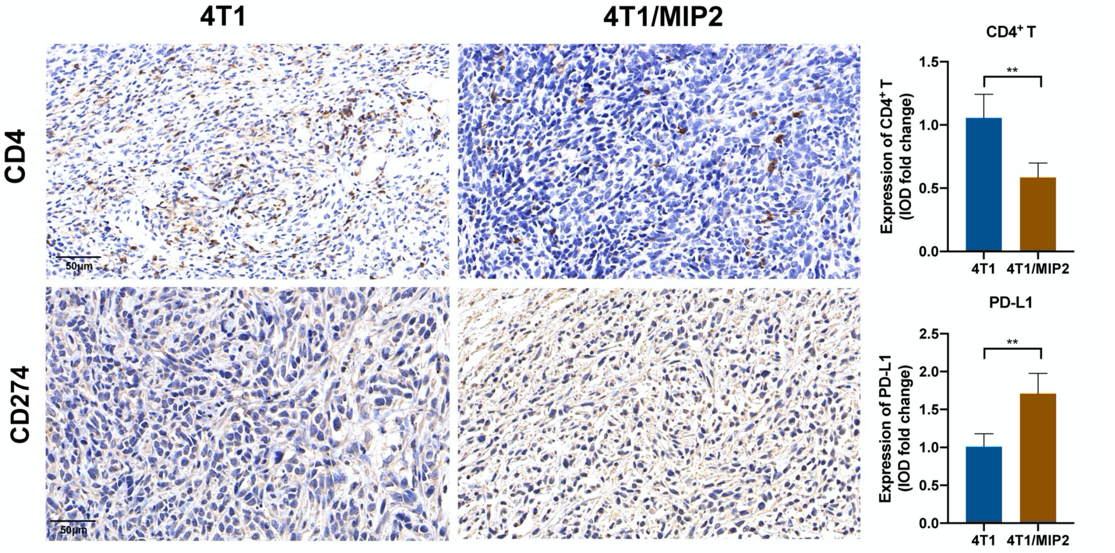

Fig. S10 Impact of MIP2 on the infiltration of CD4⁺ T cell and the expression of CD274. Representative images of IHC staining and their statistical analysis of CD4 and CD274 in the 4T1 and 4T1/MIP2 groups. IHC: immunohistochemistry; MIP2: macrophage inflammatory protein-2; PD-1: programmed cell death protein 1. Data are presented as the means ± SD of at least three independent experiments. * *P*<0.05, ** *P*<0.01, *** *P*<0.001.

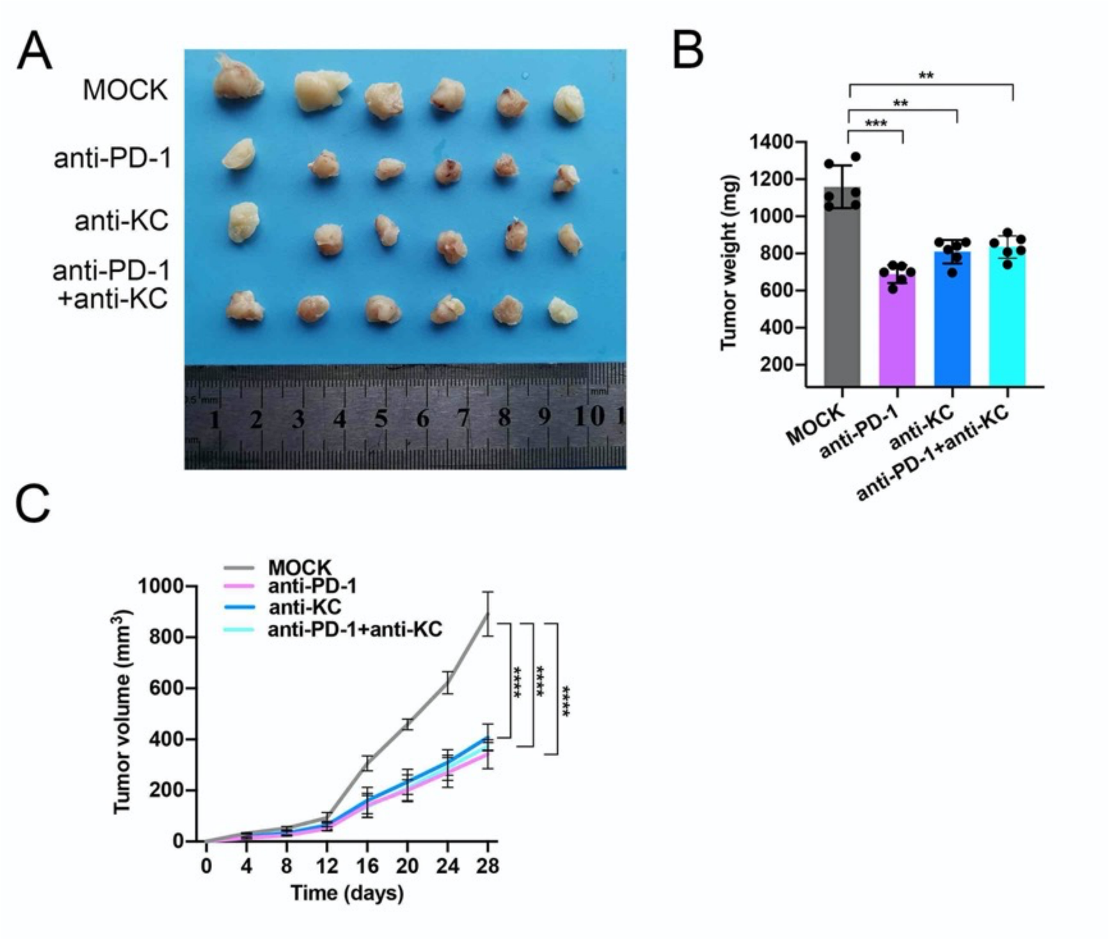

Fig. S11 Combined blockade of PD-1 and KC in TNBC. A Photo of excised subcutaneous tumors of BALB/c mice from different treatment groups. The injection of the anti-PD-1 antibody (10 μg once) and/or anti-KC antibody (10 μg once) was scheduled every two days. **B-C** Tumor weight and growth curves of mice with different treatments. PD-1: programmed cell death protein 1; KC: keratinocyte-derived chemokine; TNBC: triple-negative breast cancer.

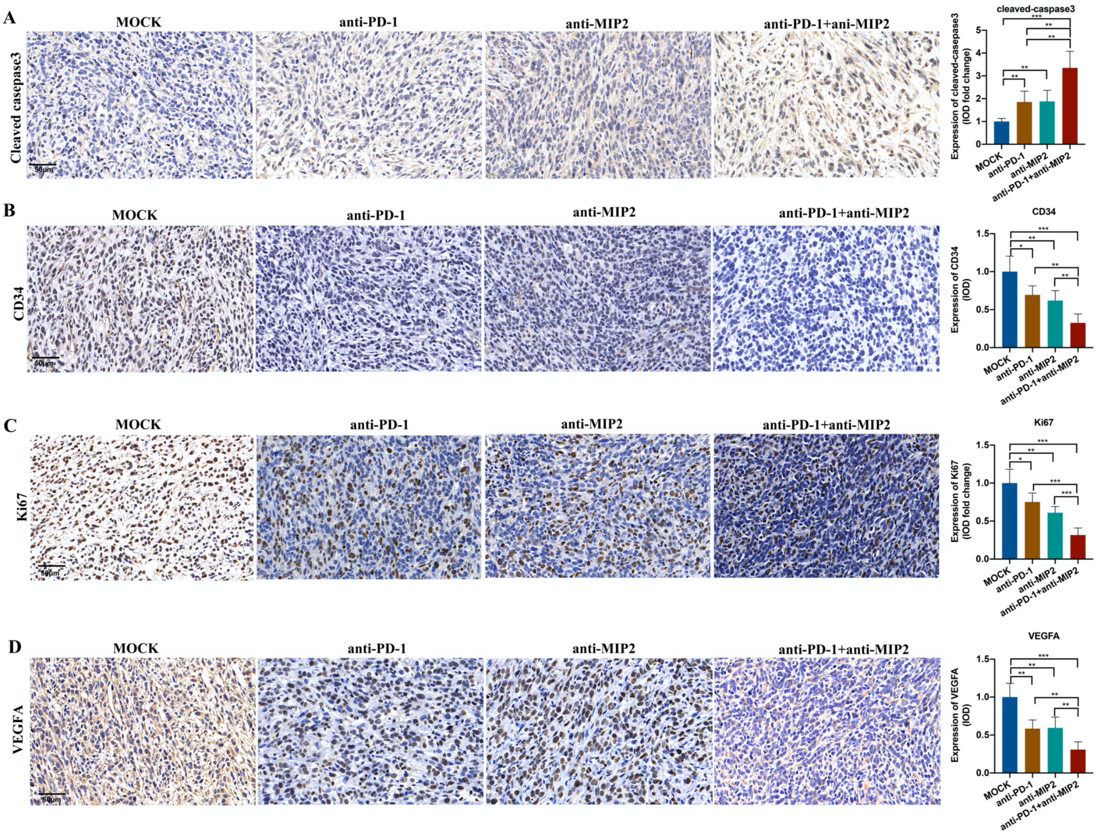

Fig. S12 Combined blockade of PD-1 and MIP2 synergistically inhibits TNBC progression by reducing cell proliferation and angiogenesis, decreasing cell stemness, and inducing cell apoptosis. A-D Representative images of IHC staining and their statistical analysis of cleaved casepase3, CD34, Ki67, and VEGFA in the MOCK, the anti-PD-1 group, the anti-MIP2 group, and the combination group. IHC: immunohistochemistry; MIP2: macrophage inflammatory protein-2; PD-1: programmed cell death protein 1; TNBC: triple-negative breast cancer. Data are presented as the means ± SD of at least three independent experiments. * P<0.05, ** P<0.01, *** P<0.001.

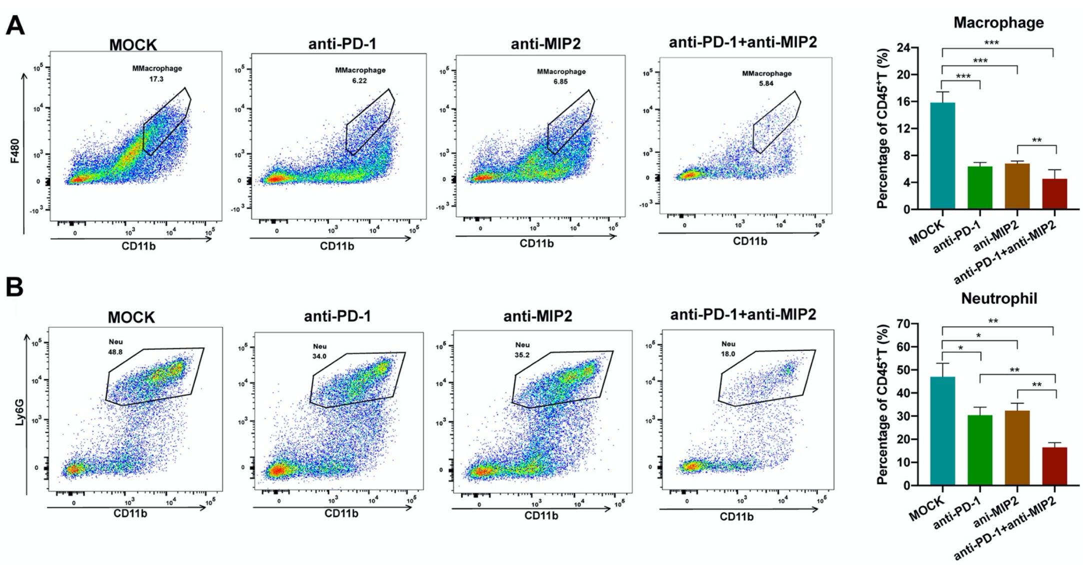

Fig. S13 Combined blockade of PD-1 and MIP2 modulates the infiltration of macrophage and neutrophil. Flow cytometry analysis of macrophage and neutrophil cells in the MOCK, the anti-PD-1 group, the anti-MIP2 group, and the combination group. MIP2: macrophage inflammatory protein-2; PD-1: programmed cell death protein 1. Data are presented as the means ± SD of at least three independent experiments. * P<0.05, ** P<0.01, *** P<0.001.

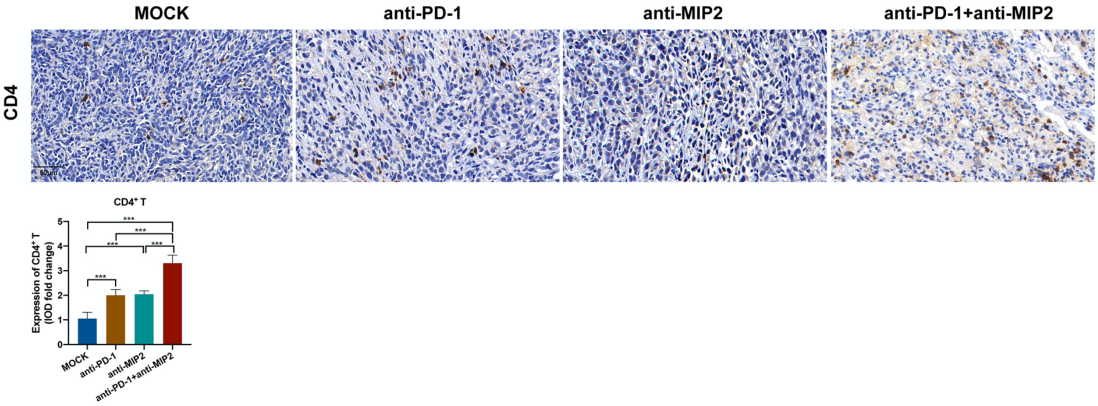

Fig. S14 Combined blockade of PD-1 and MIP2 synergistically increase the infiltration of CD4^{+} T cell. Representative images of IHC staining and their statistical analysis of cleaved CD4^{+} T cell in the MOCK, the anti-PD-1 group, the anti-MIP2 group, and the combination group. IHC: immunohistochemistry; MIP2: macrophage inflammatory protein-2; PD-1: programmed cell death protein 1. Data are presented as the means ± SD of at least three independent experiments. * P<0.05, ** P<0.01, *** P<0.001.

Table S1. Clinicopathological features of triple-negative breast cancer patients in this study.

No.	Age	Ki67	Grade	Location	Tumor size	Pathology	LVI	LN involvement	Chemotherapy	Radiotherapy
Case 1	33	80%+	III	LUOQ	9.0 cm	Metaplastic carcinoma	Yes	Yes	Yes	Yes
Case 2	46	20%+	III	RUOQ	2.0 cm	IDC	Yes	No	Yes	Yes
Case 3	39	10%+	III	LUOQ	2.9 cm	IDC	No	Yes	Yes	Yes
Case 4	50	80%+	III	RLOQ	4.5 cm	IDC with neuroendocrine differentiation	No	Yes	Yes	Yes
Case 5	59	80%+	III	LUOQ	4.5 cm	IDC	No	No	Yes	No
Case 6	64	70%+	III	RUOQ	4.0 cm	IDC	No	No	Yes	No
Case 7	56	60%+	III	LUOQ	3.5 cm	IDC	No	No	Yes	No
Case 8	45	30%+	II	RUOQ	3.0 cm	IDC with ductal carcinoma in situ	No	No	Yes	No
Case 9	56	50%+	III	LUIQ	5.5 cm	IDC with myeloid features	Yes	Yes	Yes	Yes
Case 10	61	30%+	III	LLOQ	3.4 cm	IDC with ductal carcinoma in situ	No	No	Yes	No

IDC: invasive ductal carcinoma; LLIQ: left lower inner quadrant; LLOQ: left lower outer quadrant; LN: lymph node; LUIQ: left upper inner quadrant; LUOQ: left upper outer quadrant; LVI: lymphatic vascular invasion; RLIQ: right lower inner quadrant; RLOQ: right lower outer quadrant; RUIQ: right upper inner quadrant; RUOQ: right upper outer quadrant.

Table S2. The gene list associated with immune cells in the PCR array.

Gene	1	2	3	4	5	6	7	8	9	10	11	12
**A**	BTLA	CBLB	FAS (TNFRSF6)	FOXP3	IDO1	IL15	IL2	IL2RA (CD25)	IL4	LAT	TGFB1	TNFSF14
**B**	Cd2	Cd3d	Cd3g	Cd47	Cd8a	Cd8b	Dpp4	Icam1	Irf4	Lck	Map3k7 (Tak1)	Nck1
**C**	Nr3c1	Prkcq	Prlr	Vav1	Was	Casp3	Ccnd3	Cd3e	Cxcl12	Cxcr4	IL10	IL12b
**D**	IL1b	Ptprc (Cd45)	Spp1	Tp53 (p53)	Traf6	Ada	Apc	Bad	Bcl2	Cd4	Cd74	Egr1
**E**	Flt3	Hsp90aa1	IL27	IL7	Jag2	Nos2 (Nos2a, iNos)	Pax1	Socs1	CCL3	CCL4	CCR3	CCR4
**F**	CD40	Ifng	IL18R1	IL6	CSF2	TLR4	CD200	CD200R1	CD27	CD274	CD276	CD28
**G**	CD40LG	CD70	CD80	CD86	CTLA4	HAVCR2	ICOS	ICOSLG	LAG3	PDCD1	PDCD1LG2	TNFRSF14
**H**	TNFRSF4	TNFRSF9	TNFSF4	VTCN1	NTC	NTC						

Table S3. Classification of PCR array genes according to T-cell regulators and T-cell activation.

T-Cell Regulators<br>BTLA, CBLB, FAS (TNFRSF6),<br>FOXP3, IDO1, IL15, IL2, IL2RA (CD25), IL4,<br>LAT, TGFB1, TNFSF14.	*T-Cell Activation Regulators*<br>Cd2, Cd3d, Cd3g, Cd47, Cd8a, Cd8b,<br>Dpp4, Icam1, Irf4, Lck, Map3k7 (Tak1),<br>Nck1, Nr3c1, Prkcq, Prlr, Vav1, Was.
T-Cell Proliferation<br>Casp3, Ccnd3, Cd3e, Cxcl12, Cxcr4, Il10, Il12b,<br>Il18, Il1b, Ptprc (Cd45), Spp1, Tp53 (p53), Traf6.	*T-Cell Differentiation*<br>Ada, Apc, Bad, Bcl2, Cd4, Cd74, Egr1, Flt3, Hsp90aa1,<br>Il27, Il7, Jag2, Nos2, Pax1, Socs1.
T-Cell Polarization<br>Ccl3 (Mip-1a), Ccl4 (Mip-1b), Ccr3, Ccr4.	*Th1 & Th2 Differentiation*<br>Cd40, Ifng, Il18r1, Il6.
Th1 & Th2 Developmental Regulators<br>Cd2, Csf2, Tlr4.	CD200, CD200R1, CD27, CD274, CD276, CD28, CD40LG, CD70, CD80, CD86, CTLA4,<br>HAVCR1, HAVCR2, ICOS, ICOSLG, LAG3, PDCD1, PDCD1LG2, TNFRSF14, TNFRSF4,<br>TNFRSF9, TNFSF4, VTCN1.

Table S4. Primary antibodies for western blot, immunofluorescence, immunohistochemistry, and flow cytometry.

Protein	Concentration Western blot	Concentration IF	Concentration IHC	Concentration Flow cytometry	Specificity	Company Lot No.
Adiponectin	/	1:1000	/	/	Rat anti-human	Abcam Cat#181281
Adiponectin	/	/	1:50	/	Rat anti-human	Novus Biologicals Cat# NBP2-22450
FABP4	/	1:1000	1:50	/	Rat anti-human	Abcam Cat# ab92501
Perilipin	/	1:1000	1:50	/	Rat anti-human	Abcam Cat# ab172907
CD4	/	1:100	1:100	/	Rat anti-mouse	Novus Biologicals Cat#NBP1-19371
CD4	/	/	/	1:100	Rat anti-mouse	BD Biosciences Cat#563331
CD8	/	1:100	1:100	/	Rat anti-mouse	Novus Biologicals Cat#NBP1-49045
CD8	/	/	/	1:100	Rat anti-mouse	BD Biosciences Cat#568163
CTLA4	/	1:50	1:50	/	Rat anti-mouse	Santa Cruz Cat#sc-376016
PD-L1	/	1: 100	/	/	Rat anti-mouse	Novus Biologicals Cat# NBP1-76769
PD-L1	/	/	1:100	/	Rat anti-mouse	Novus Biologicals Cat# NBP1-76769
CD11b	/	/	/	1:100	Rat anti-mouse	BD Biosciences Cat#741242
Ly6G	/	/	/	1:100	Rat anti-mouse	BD Biosciences Cat#746614
CD45	/	/	/	1:100	Rat anti-mouse	BD Biosciences Cat# 753633
E-cadherin	1:1000	1:1600	1:400	/	Rat anti-mouse or human	CST Cat#3195
N-cadherin	1:1000	1:200	1:125	/	Rat anti-mouse or human	CST Cat#13116
Vimentin	1:1000	1:100	1:100	/	Rat anti-mouse or human	CST Cat#5741
Snail	1:1000	/	/	/	Rat anti-mouse or human	CST Cat#3879

CXCL8	1:1000	/	/	/	Rat anti-human	CST Cat#94407
CXCL8	/	1 μg/ml	1:100	/	Rat anti-human	Novus Biologicals Cat#NBP2-33819
AKT	1:1000	/	/	/	Rat anti-mouse or human	CST Cat#9272
p-AKT	1:1000	/	/	/	Rat anti-mouse or human	CST Cat#4060
GAPDH	1:1000	/	/	/	Rat anti-mouse or human	CST Cat# 5174

CXCL8: C-X-C motif chemokine ligand; IF: immunofluorescence; IHC: immunohistochemistry.

Table S5. Primers for RT-qPCR applied in this study.

Gene name	Forward sequence	Reverse sequence
**CXCL8**	AGAGTGGACCACACTGCGC	ACATCCCAACGGTCTACGTTA
**E-cadherin**	CTGAGAACGAGGCTAACG	TTCACATCCAGCACATCC
**N-cadherin**	GCGTCTGTAGAGGCTTCTGG	GCCACTTGCCACTTTTCCTG
**Vimentin**	CCTATGTGACCCGGTCCTCG	AAGGTCAAGACGTGCCAGAG
**Snail**	CACCTCCAGACCCACTCAGAT	CCTGAGTGGGGTGGGAGCTTCC
**Twist**	CCACGCTGCCCTCGGACAAG	CCAGGCCCCCTCCATCCTCC
**AKT**	AGCGACGTGGCTATTGTGAAG	GCCATCATTCTTGAGGAGGAAGT
**STAT3**	TGAGACTTGGGCTTACCATTGGGT	TCTTTAATGGGCCACAACAGGGCT
**PI3K**	TAT TTGGACTTTGCGACAAGACT	TCG AACGTACTGGTCTGGATAG
**GAPDH**	ACCCACTCCTCCACCTTTGA	CTGTTGCTGTAGCCAAATTCGT

CXCL8: C-X-C motif chemokine ligand; RT-qPCR: Real-time quantitative polymerase chain reaction.
