# Supplementary material for: Targeting cancer-associated adipocyte-derived CXCL8 inhibits triple-negative breast cancer progression and enhances the efficacy of anti-PD-1 immunotherapy
Source: Cell Death Dis. 2023 Oct 28;14(10):703. doi: 10.1038/s41419-023-06230-z (PMC10613226; doi:10.1038/s41419-023-06230-z)
Supplement: Supplementary file 2 — Supplemental file_Uncropped WB [file 41419_2023_6230_MOESM2_ESM.pdf]

Original data files of uncropped Western blots for Supplementary

Figure 2I

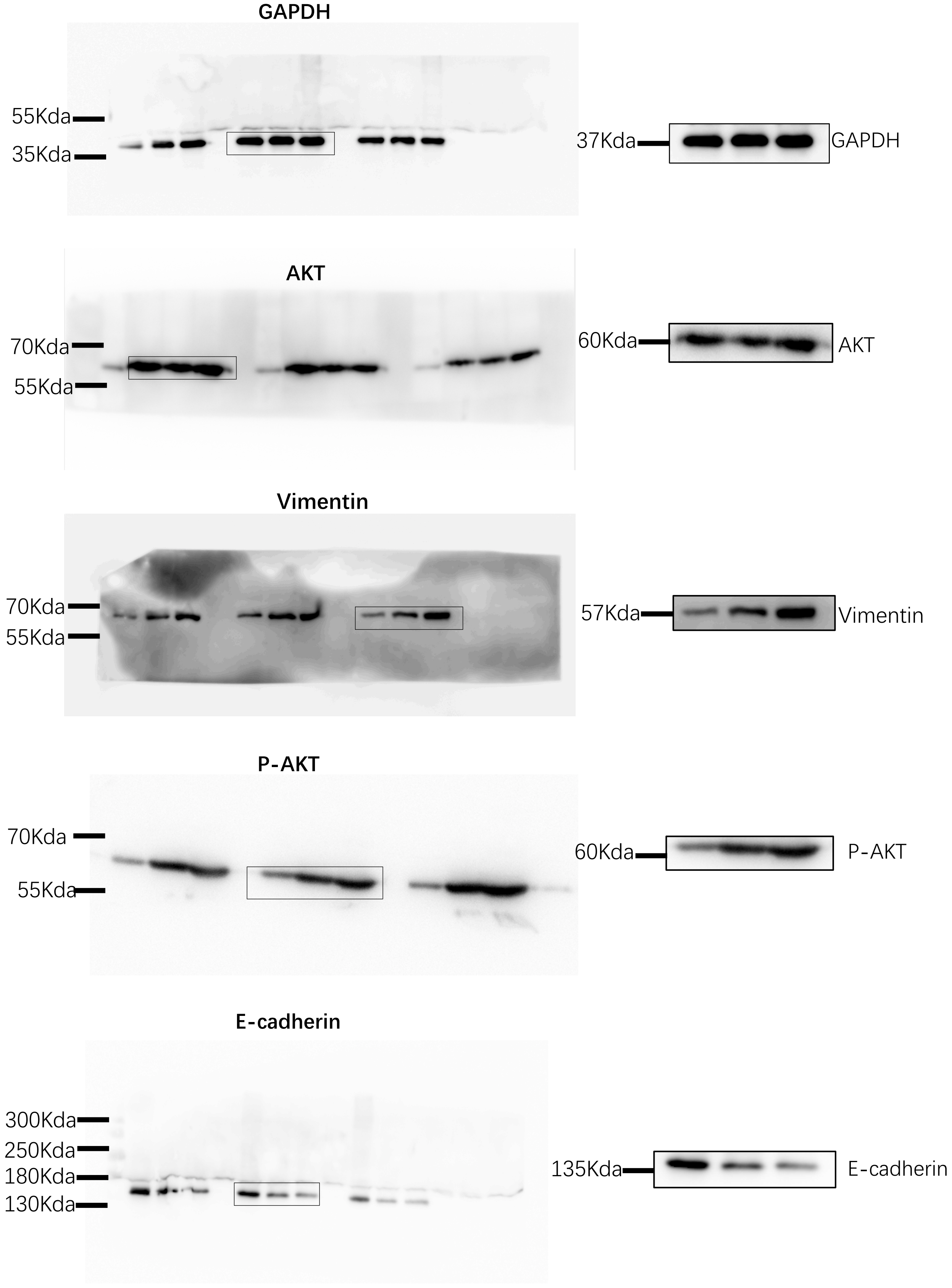

N-cadherin

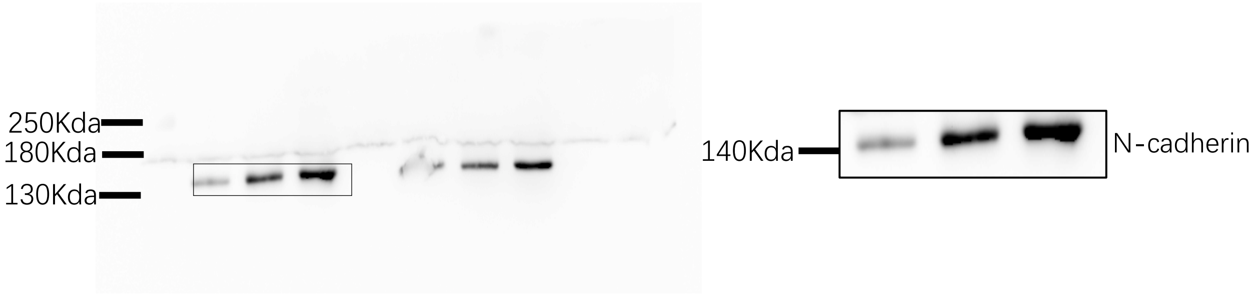

Snail

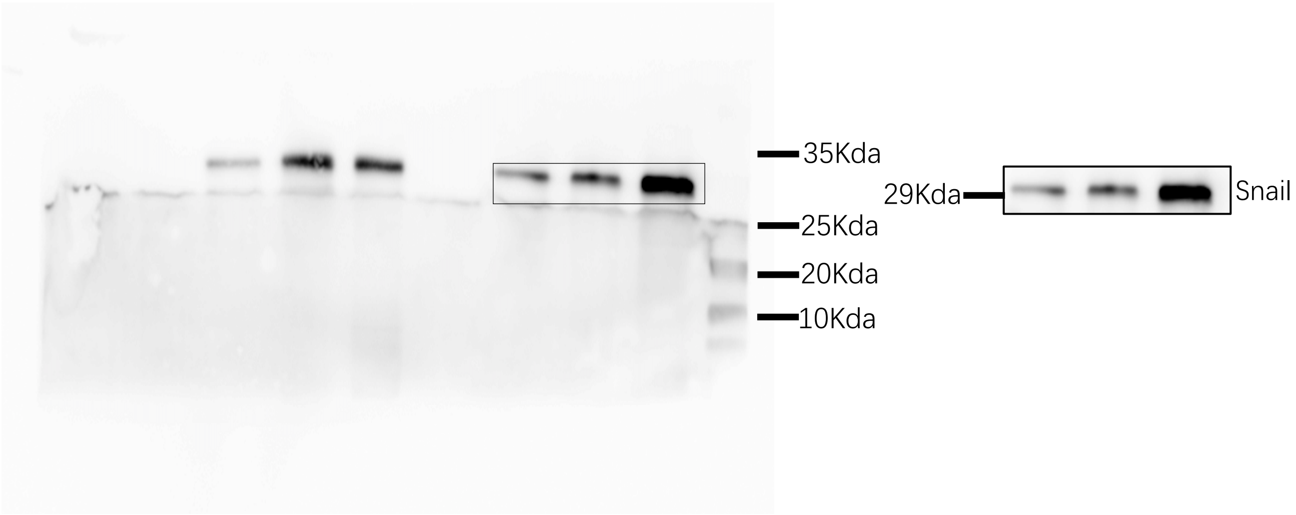

Original data files of uncropped Western blots for Supplementary Figure 4N

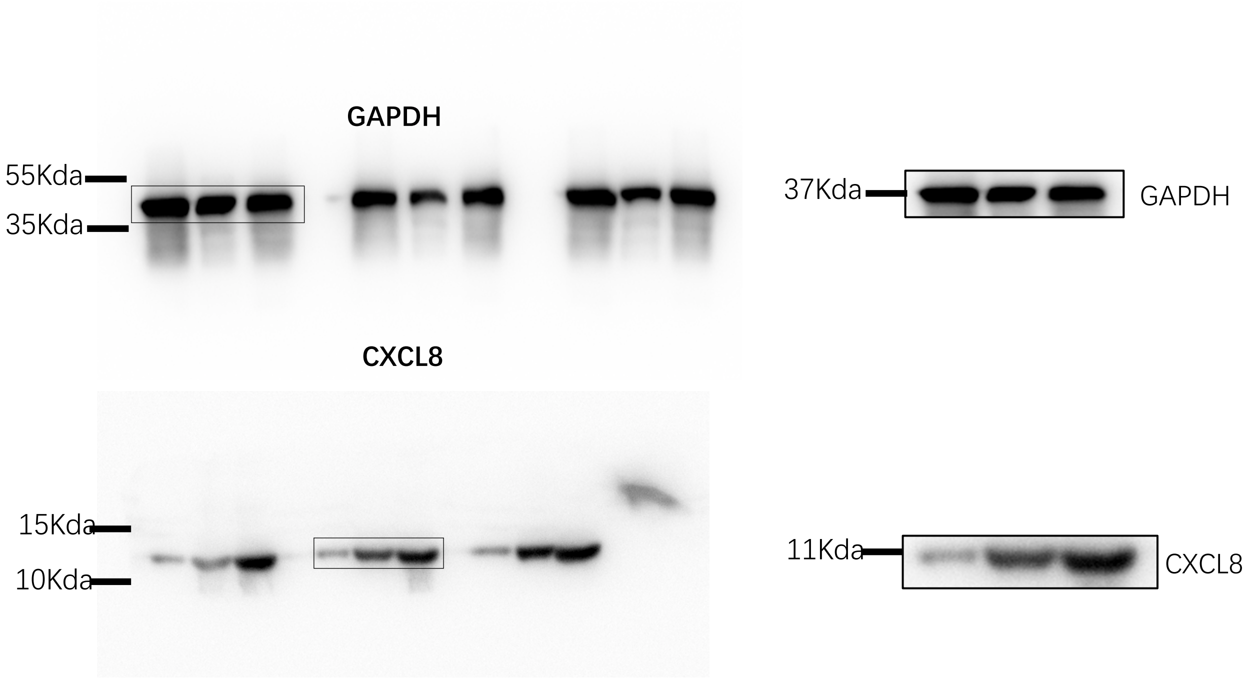

Original data files of uncropped Western blots for Supplementary Figure 5G, I

AKT

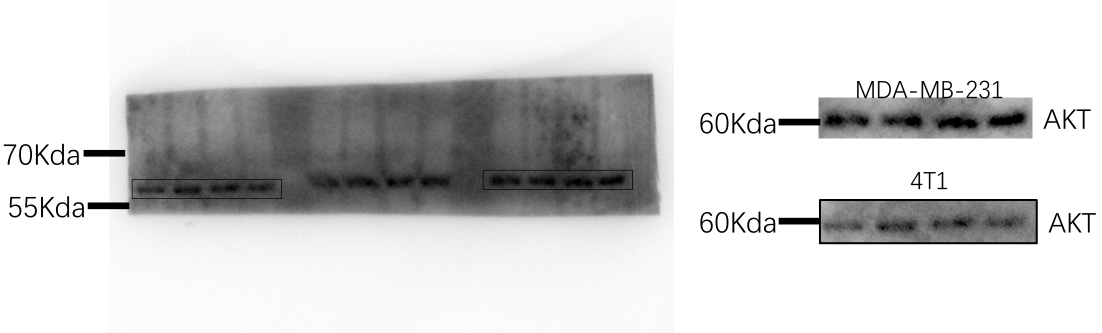

E-cadherin

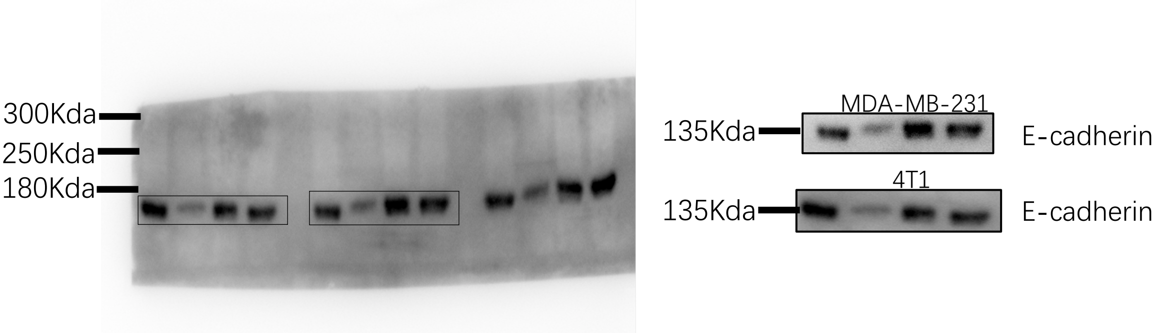

Snail

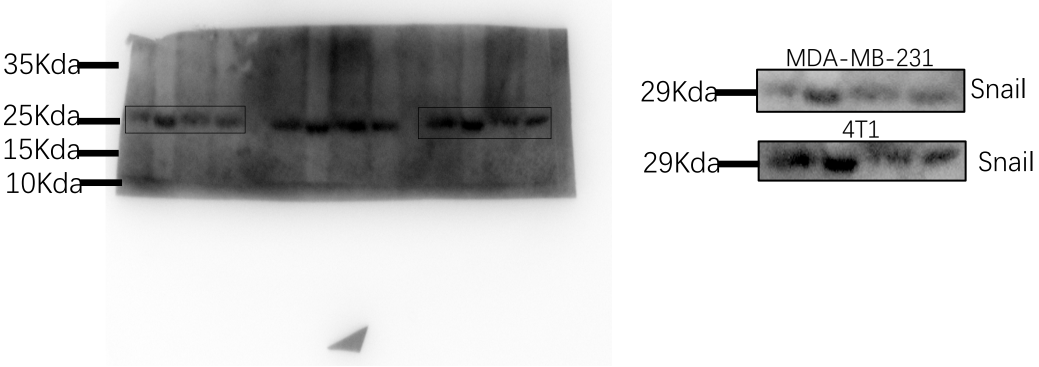

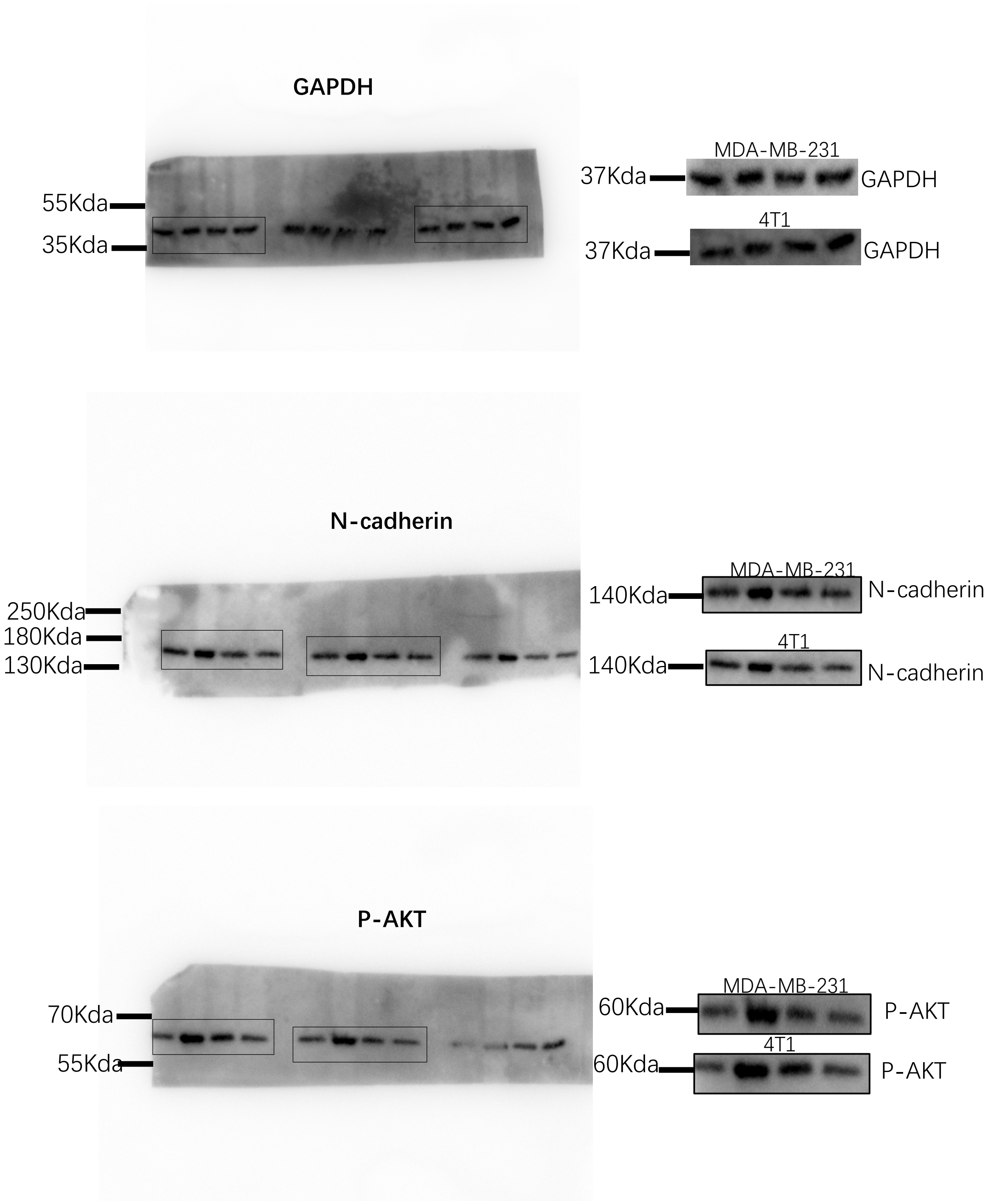
GAPDH
55Kda
35Kda
MDA-MB-231
37Kda
GAPDH
4T1
37Kda
GAPDH
N-cadherin
250Kda
180Kda
130Kda
MDA-MB-231
140Kda
N-cadherin
4T1
140Kda
N-cadherin
P-AKT
70Kda
55Kda
MDA-MB-231
60Kda
P-AKT
4T1
60Kda
P-AKT

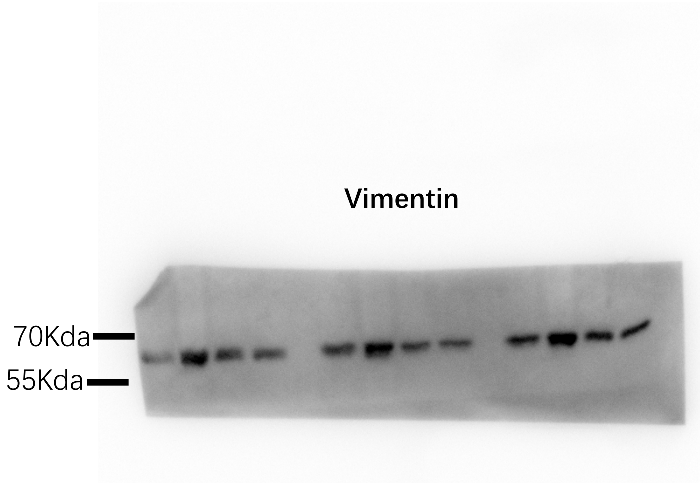
Vimentin
70Kda
55Kda

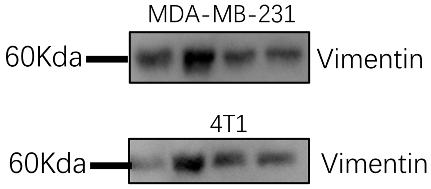
MDA-MB-231
60Kda
Vimentin
4T1
60Kda
Vimentin
